# Two distinct alcohol dehydrogenase classes mediate bacterial 2‐hydroxyethylphosphonate catabolism

**DOI:** 10.1002/pro.70783

**Published:** 2026-09-15

**Authors:** Francesca Piazza, Marianna Rodella, Nicholas Salanti, Giovanni Merici, Elisa Rossi, Claudio Rivetti, Florian Götzinger, Katharina Pallitsch, Alessio Peracchi

**Affiliations:** ^1^ Department of Chemistry, Life Sciences and Environmental Sustainability University of Parma Parma Italy; ^2^ Institute of Organic Chemistry University of Vienna Vienna Austria

**Keywords:** alcohol dehydrogenases, bacterial metabolism, phosphonates, phosphorus mineralization

## Abstract

Phosphonates are molecules containing a direct carbon‐phosphorus bond. In nature, these compounds are produced by a variety of organisms, and many environmental microbes are able to degrade specific phosphonates and feed on them. This work focuses on 2‐hydroxyethylphosphonate (HEP)—a natural compound that is found frequently in the environment, but whose microbial catabolism has remained poorly characterized. Starting from genomic analyses, we report here that specific pathways for HEP degradation in bacteria do exist and rely on the oxidation of HEP into the activated compound phosphonoacetaldehyde (PAA), which can be further processed by known hydrolases. The key conversion of HEP to PAA can be carried out by two unrelated groups of dehydrogenases: one comprises homologs of the biosynthetic enzyme PhpC (PAA reductase), whereas the other consists of newly described enzymes that we termed PbfG. Despite sharing the same substrates and using Zn^2+^ as the metal cofactor, PhpC‐type and PbfG‐type dehydrogenases exhibit markedly different structural and catalytic features, outlining a clear example of convergent evolution. However, PbfG enzymes are catalytically more efficient and apparently more specialized for HEP degradation. Overall, our results reveal the ample distribution in bacteria of specific routes for HEP catabolism. Furthermore, although the degradation pathways for HEP and of 2‐aminoethylphosphonate (AEP; the most common natural phosphonate) share some enzymes, the corresponding gene clusters are often distinct, suggesting a selective advantage in keeping the two catabolic processes separate.

## 1 INTRODUCTION

Organophosphonates (henceforth phosphonates for brevity) are organic compounds that contain a direct carbon‐phosphorus bond (Horsman & Zechel, 2017). They occur in the environment, both as natural products (Kafarski, 2019) and as anthropogenic pollutants (Furtak et al., 2024). Many microorganisms are able to degrade these compounds, primarily to retrieve phosphorus, but also, not infrequently, to obtain energy, carbon, and/or nitrogen (Chin et al., 2016; Fox & Mendz, 2006; Ruffolo et al., 2023; Sosa et al., 2019).

Some important pathways for phosphonate degradation have been characterized at the biochemical and molecular level and can be schematically distinguished based on the chemistry of the C−P bond cleavage—either a radical, oxidative or hydrolytic reaction (Horsman & Zechel, 2017; Martinez et al., 2010). A radical mechanism is operated by the C−P‐lyase system, a multiprotein complex composed of five or more distinct subunit types, which can degrade a variety of phosphonates to scavenge phosphorus (the complex is typically expressed only under severe phosphorus starvation) (Manav et al., 2018). In comparison, hydrolytic and oxidative pathways are generally simpler, act on narrower substrate ranges, and can support the acquisition of nutrients beyond phosphate (Pallitsch & Zechel, 2023; Ruffolo et al., 2023).

**FIGURE 1:**
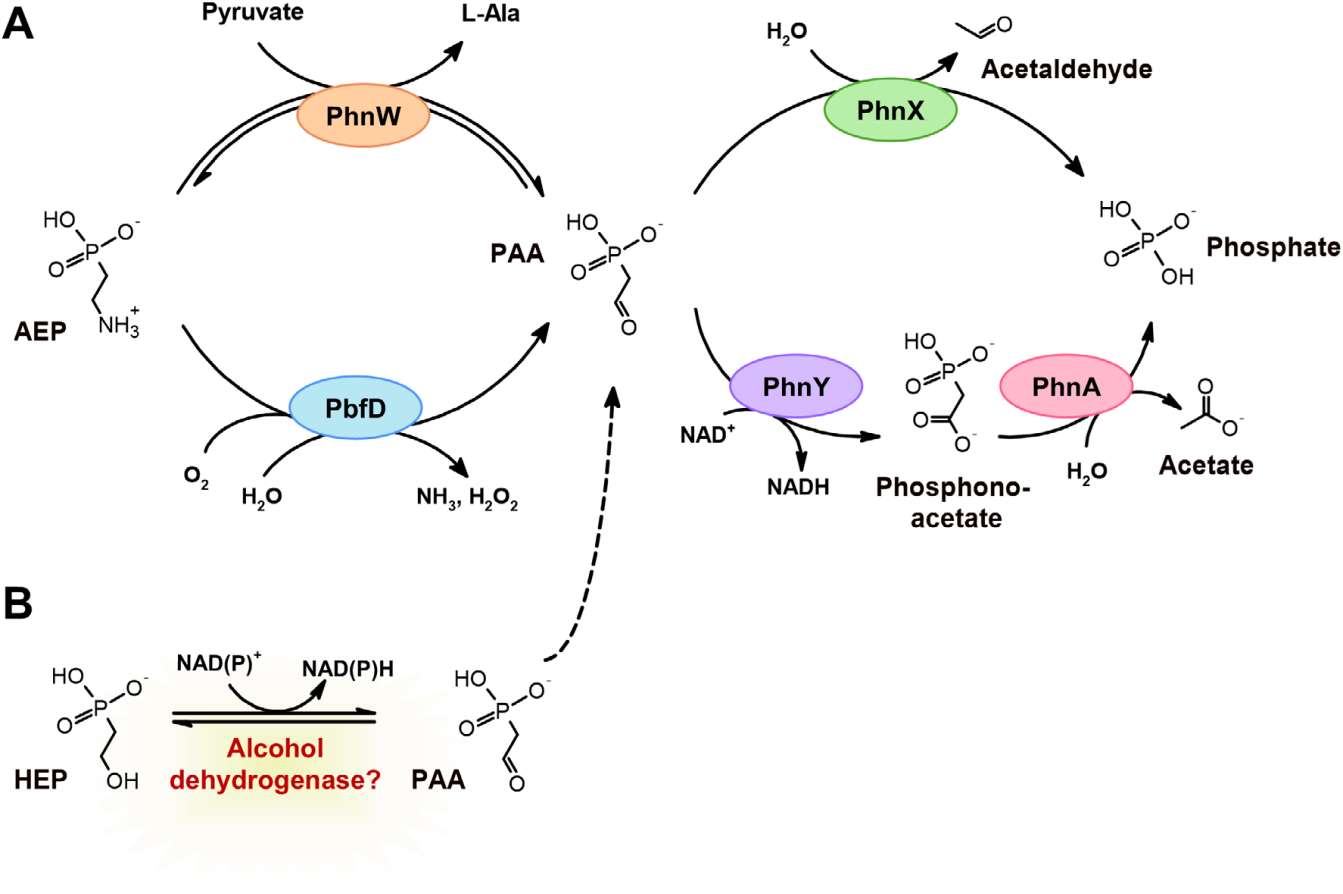
Hydrolytic pathways for phosphonate catabolism. (a) ‘Superpathway’ for the hydrolytic AEP degradation. The common intermediate PAA can be produced from AEP by a reversible transamination or by an irreversible oxidative deamination (Zangelmi et al., 2023). PAA can then be further processed by the hydrolase PhnX (to yield phosphate and acetaldehyde) (Dumora et al., 1989) or by the consecutive reactions of the enzymes PhnY and PhnA (to yield phosphate and acetate) (Kim et al., 2011; McGrath et al., 1995). (b) A hypothetical alcohol dehydrogenase, oxidizing HEP to PAA, could enable HEP degradation through the hydrolytic pathways.

In particular, many bacteria possess specialized ‘hydrolytic’ pathways for the breakdown of 2‐aminoethylphosphonate (AEP; also known as ciliatine), which is the most widespread biogenic phosphonate (Kafarski, 2019; Li & Horsman, 2022). The hydrolytic pathways for AEP degradation are an intertwined set of reactions that share the starting point (AEP), one of the final products (inorganic phosphate), and the activated intermediate, phosphonoacetaldehyde (PAA). However, they may encompass completely different sets of enzymes in different organisms (Figure 1a). The conversion of AEP into PAA is most typically carried out by the transaminase PhnW, but it can also be accomplished by the amine oxidase PbfD (Zangelmi et al., 2023). PAA can be further processed by different enzymes, with the simplest route involving its direct cleavage into acetaldehyde and inorganic phosphate by the enzyme PhnX (Dumora et al., 1989). Otherwise, PAA can be oxidized by the dehydrogenase PhnY to phosphonoacetate, which is then cleaved by the hydrolase PhnA (Figure 1a) (Kim et al., 2011; McGrath et al., 1995).

2‐Hydroxyethylphosphonate (HEP) is another naturally occurring compound, whose environmental abundance may be substantial (Li & Horsman, 2022). In particular, HEP is an intermediate in the biosynthesis of methylphosphonate, carried out by a number of marine prokaryotes (Born et al., 2017; Metcalf et al., 2012). HEP was also identified as an integral intermediate in the biosynthesis of various bioactive substances, produced by different *Streptomyces* species (Blodgett et al., 2007; Shao et al., 2008; Zhang et al., 2022). Furthermore, it was found incorporated in secondary metabolites produced by the cyanobacterium *Aphanizomenon flos‐aquae* (Kaya et al., 2006) and by some sponges (Kim et al., 2013), as well as in phosphonoglycans from two actinomycetes (Yu et al., 2014). Finally, HEP in the environment could also derive from the spontaneous degradation of Ethephon (2‐chloroethylphosphonic acid), a man‐made chemical used as a plant growth regulator (Lantz & Casida, 2013).

Up to date, the only documented catabolic fate of HEP involved its conversion to ethylene by the ‘aspecific’, phosphate starvation‐induced C−P lyase complex (Repeta et al., 2016). However, in principle, the degradation of HEP could be easily connected to the hydrolytic pathways of AEP breakdown. In fact, oxidation of the alcohol function of HEP by some NAD(P)‐dependent dehydrogenase would directly generate PAA, which could then be processed by PhnX or by the PhnY‐PhnA enzymes (Figure 1b).

NAD‐dependent enzymes that interconvert HEP and PAA have already been described in *Streptomyces* species (Shao et al., 2008). Such enzymes serve to *synthesize* HEP, within biochemical pathways for the production of secondary metabolites: phosphinothricin tripeptide in *Streptomyces viridochromogenes* (Blodgett et al., 2007), argolaphos in *S. monomycini* (Zhang et al., 2022), fosfomycin in *S. wedmorensis* and dehydrophos in *S. luridus* (Shao et al., 2008). These enzymes exhibit rather divergent sequences and have been assigned different names (PhpC, AglG, FomC, and DhpG, respectively). However, they all belong to the same structural family and catalyze the very same reaction; so here, for simplicity, we use the term PhpC to refer to all of them.

Could PhpC‐type enzymes function in HEP degradation, as well as in its biosynthesis? Indeed, in a genomic and metagenomic survey of phosphonate‐related microbial gene clusters, Lockwood and coworkers reported that <50% of the *phpC* genes were not apparently connected to phosphonate production, as they did not cluster with the biosynthetic genes *pepM* and *aepY* (Lockwood et al., 2022). The authors therefore suggested that these *phpC* homologs could play a catabolic role, providing some examples of *phpC* genes located within operons dedicated to AEP degradation (i.e., containing *phnW* together with either *phnX* or the *phnY‐phnA* couple) (Lockwood et al., 2022).

Herein, we expand the analysis by Lockwood et al. by studying in detail the non‐biosynthetic clusters that contain *phpC* homologs. We show that many such clusters, while evidently degradative, are *not* dedicated to AEP catabolism. We further show that genes for a second, very different alcohol dehydrogenase (which we termed PbfG) occur in other degradative clusters, also often not dedicated to AEP breakdown. Biochemical characterization of one representative for each group of dehydrogenases confirmed that both PhpC and PbfG efficiently oxidize HEP, albeit with very distinct catalytic features. Overall, our results indicate that the routes for specific HEP catabolism are widespread in several bacterial phyla and are similar in strategy while using different enzymes.

## 2 RESULTS

### 2.1 An analysis of degradative gene clusters containing *phpC*

We conducted an extensive search of bacterial genomes, looking for *phpC* homologs not included in biosynthetic operons, and analyzed their genomic context. We found apparently degradative clusters encompassing *phpC‐*like sequences mostly in Pseudomonadota (Alpha‐ and Gammaproteobacteria) and in Bacillota, but also in Fibrobacterota and Planctomycetota (Table S1 and Figure S1).

**FIGURE 2:**
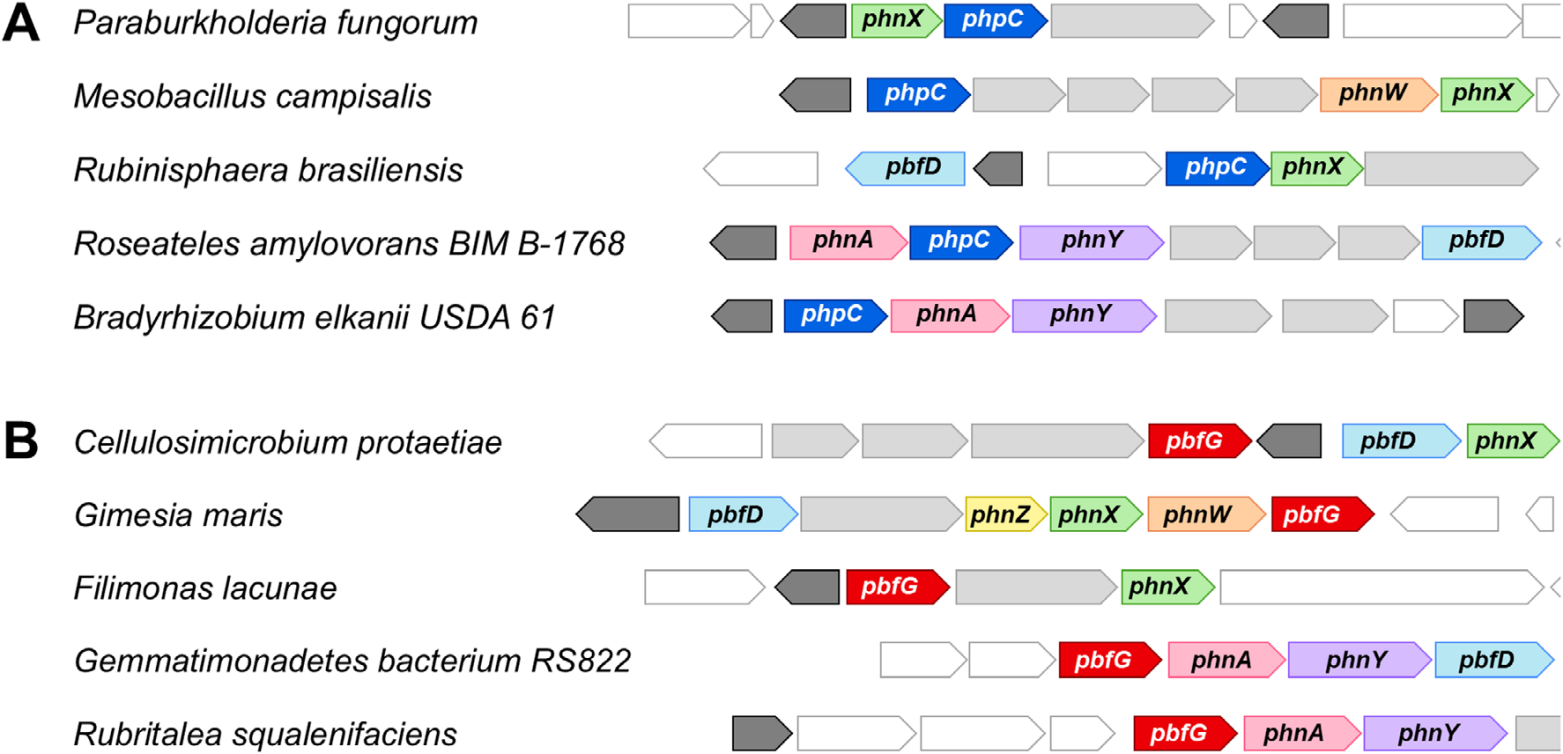
Examples of clusters dedicated to phosphonate degradation harboring genes for potential HEP dehydrogenases. (a) Genes encoding PhpC homologs (pf00465) are shown in blue; the accession IDs of the encoded proteins are reported in Table S1. Other highlighted genes include *phnX* (green), *phnW* (orange), *pbfD* (azure), *phnA* (pink) and *phnY* (purple), Putative transporter genes are shown in light gray, predicted transcription regulators are in dark gray. (b) Genes encoding PbfG, a pf00107 alcohol dehydrogenase are shown in red; the corresponding accession IDs are provided in Table S2. The *phnZ* gene (yellow) is involved in the degradation of (*R*)‐2‐amino‐1‐hydroxyethylphosphonate (Horsman & Zechel, 2017; Pallitsch & Zechel, 2023). Other genes are colored as in panel “a”.

Of note, these *phpC* homologs seldom occurred in clusters evidently dedicated to AEP catabolism (i.e., clusters containing *phnW* or *pbfD*, together with *phnX* or the *phnY‐phnA* couple); more often, the *phpC* genes were associated with just *phnX* or *phnY‐phnA* (Table S1 and Figure 2a). This gene composition is consistent with clusters specifically dedicated to HEP degradation.

### 2.2 Identification of PbfG, a second potential HEP dehydrogenase unrelated to PhpC

We then expanded our analysis by searching for other genes annotated as alcohol dehydrogenases that might be recurrently associated with PAA‐degrading genes. This search identified a candidate (whose product belongs to Pfam pf00107, Zinc‐binding dehydrogenase) that, despite being apparently unrelated to *phpC*, often occurred in degradative clusters with gene compositions strongly reminiscent of the pattern described above. That is, the clusters with the pf00107 gene encompassed *phnX* or the *phnY‐phnA* duo but in several cases lacked *phnW* or *pbfD*. Based on the consistent association of this dehydrogenase gene with genes for PAA degradation and following the nomenclature adopted previously (Ruffolo et al., 2025; Zangelmi et al., 2021, 2023), we termed the encoded protein “phosphonate breakdown factor G” (PbfG).

Degradative clusters containing *pbfG* were found in Actinomycetota (particularly among the Microbacteriales and Mycobacteriales but also in other orders), Planctomycetota (Pirellulales, Cyanobacterales, and others), Bacterioidota (particularly in the order Cytophagales, which are known as important remineralizers), Gemmatimonadota, and Verrucomicrobiota. A representative list of PbfG proteins and their corresponding gene clusters is provided in Table S2. Examples of different types of gene clusters encompassing *pbfG* are shown in Figure 2b.

While at the protein level the most similar enzymes of known function were threonine dehydrogenases (PbfG from *Cellulosimicrobium protaetiae* showed 32.5% sequence identity to threonine dehydrogenase from *Escherichia coli* [Aronson et al., 1989]), the similarities between the degradative gene clusters encompassing *phpC* (Table S1 and Figure 2a) and *pbfG* (Table S2 and Figure 2b), plus the occasional finding of *pbfG* in apparently biosynthetic clusters (in *Actinoplanes*; Figure S2) suggested that PhpC and PbfG could fulfill the same functional role. In other words, the data suggested that PbfG could function as a HEP dehydrogenase (or PAA reductase).

### 2.3 Probing the HEP dehydrogenase activity of PhpC and PbfG

Genes encoding PhpC from *Paraburkholderia fungorum* and PbfG from *C. protaetiae* were chemically synthesized and cloned into bacterial expression vectors. The genes were expressed in *E. coli* and the recombinant His‐tagged proteins were purified as described in the Methods. Size exclusion chromatography yielded apparent molecular masses of ~78 kDa for PhpC and ~64.2 kDa for PbfG (Figure S3), suggesting that both proteins exist in solution as dimers (the calculated monomeric masses of PhpC and PbfG are 36.7 and 38.5 kDa, respectively).

**FIGURE 3:**
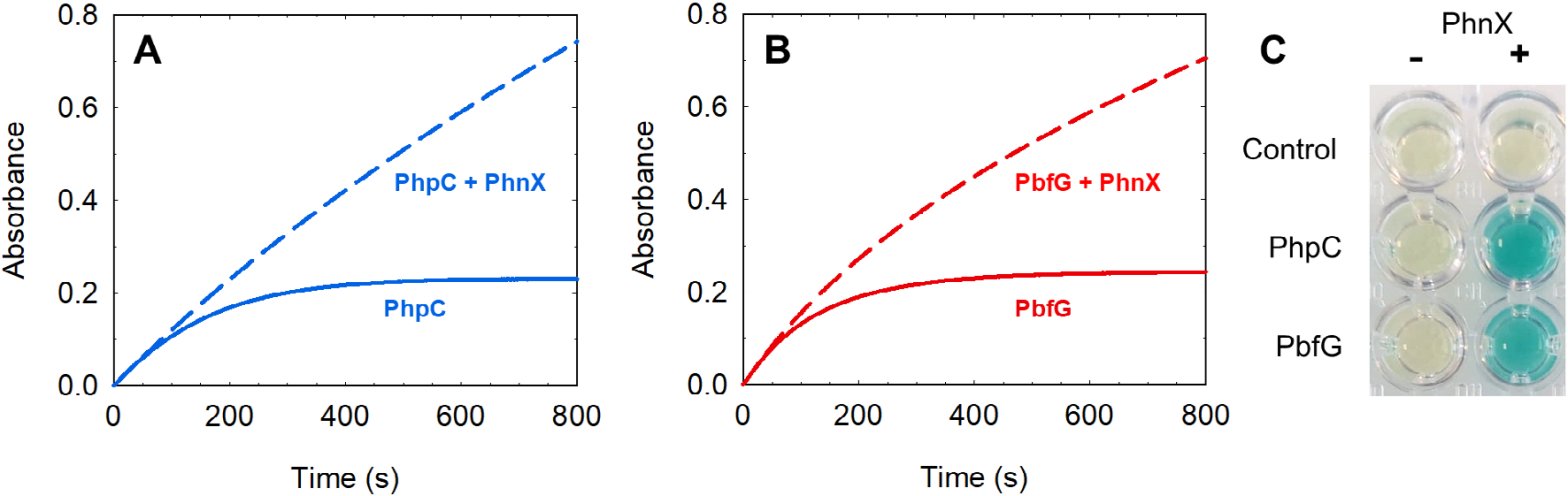
PhpC and PbfG can both oxidize HEP to PAA (a) Reaction of PhpC (2 μM) with HEP (1 mM) and NAD^+^ (1.5 mM) in the absence and in the presence of PhnX (1 μM). Other conditions: 50 mM TEA‐HCl (pH 8.0) 1 mM MgCl_2_ and 1 mM β‐mercaptoethanol. (b) Reaction of PbfG (0.025 μM) with HEP (1 mM) and NAD^+^ (1.5 mM) ± PhnX (1 μM); other conditions as in panel A, except that 0.1% of BSA was added to stabilize PbfG at the low concentration used in this assay. (c) BiomolGreen assay, revealing the formation of phosphate from HEP in the presence of either dehydrogenase plus PhnX. The concentrations of substrates and (when indicated) enzymes were as in panels A and B. Reactions were conducted for 4 h at room temperature, after which 15 μL of each reaction mixture were added to 200 μL of BIOMOL® Green reagent.

We then tested the purified proteins for the ability to oxidize HEP using NAD^+^ as the electron acceptor. Both PhpC and PbfG catalyzed the reaction with appreciable efficiency, leading to the formation of NADH (Figure 3a,b). While the two enzymes showed different catalytic efficiencies, their reactions reached identical endpoints, where (despite the excess of HEP) only a fraction of the initial NAD^+^ had been reduced. This was consistent with the equilibrium of the reaction favoring the reagents (NAD^+^ and HEP) with respect to the products (NADH and PAA). However, when the reaction was carried out in the presence of the hydrolase PhnX, which consumes PAA, the accumulation of NADH continued beyond the endpoint (Figure 3a,b). Moreover, when PhnX was present, inorganic phosphate was eventually formed (Figure 3c).

### 2.4 Differential properties of PhpC and PbfG

These results confirmed the initial hypothesis that PhpC and PbfG can both serve to catabolize HEP. Despite being isofunctional, however, the two enzymes showed markedly distinct enzymological properties.

First, PhpC appeared inherently less active than PbfG; for example, while very similar kinetics can be observed in Figure 3a,b, the PhpC concentration in panel 3A was 80‐fold higher than the PbfG concentration in panel 3B.

**FIGURE 4:**
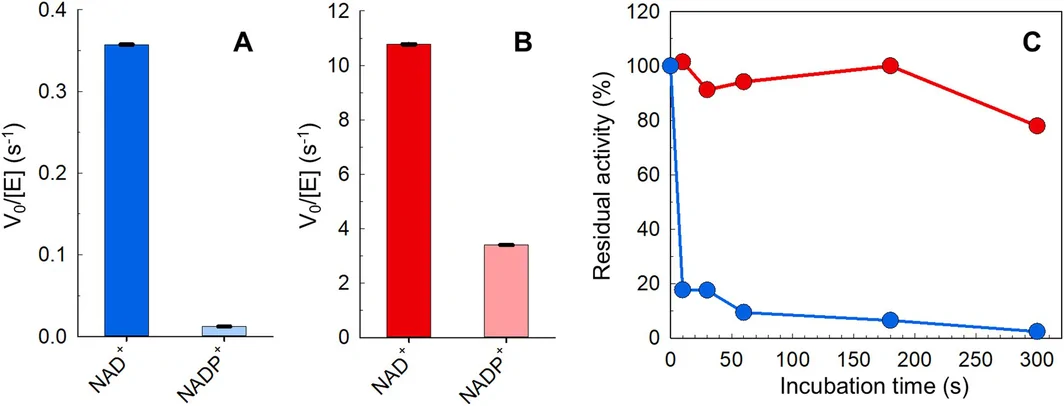
Distinct functional properties of PhpC and PbfG (a) Relative activity of PhpC (0.33 μM) in NAD^+^/NADP^+^. Conditions: NAD^+^/NADP^+^ 1.5 mM (controls established that such a concentration was saturating) and 5 mM HEP. (b) Relative activity of PbfG (0.1 μM) in NAD^+^/NADP^+^; conditions as in panel A. (c) Residual activity of PhpC (blue) and PbfG (red) as a function of the incubation time in the presence of 0.05 mM EDTA.

Second, PhpC had a strong preference for NAD^+^ as a cofactor, showing very low activity in the presence of NADP^+^ (Figure 4a). In contrast, PbfG, while still preferring NAD^+^, was able to use NADP^+^ with just a threefold lower efficiency (Figure 4b).

Finally, PhpC activity decreased rapidly in the presence of sub‐millimolar concentrations of ethylenediaminetetraacetic acid (EDTA; Figure 4c) suggesting that the enzyme activity depends on a rather weakly bound metal ion. In contrast, PbfG was only very modestly inhibited by EDTA even after 5 min of incubation (Figure 4c).

### 2.5 Reactivation of EDTA‐treated PhpC by different divalent metal ions

While pf00465 (to which PhpC belongs) is annotated as “iron‐containing dehydrogenase family”, the biosynthetic PhpC from *S. viridochromogenes* was reportedly zinc‐dependent (Shao et al., 2008) and so was the related enzyme sulfoacetaldehyde reductase from *Bifidobacterium kashiwanohense* (Zhou et al., 2019) whereas other PhpC homologs are activated by or contain a variety of divalent metal ions (e.g. Elleuche et al., 2014; Qi et al., 2016).

**FIGURE 5:**
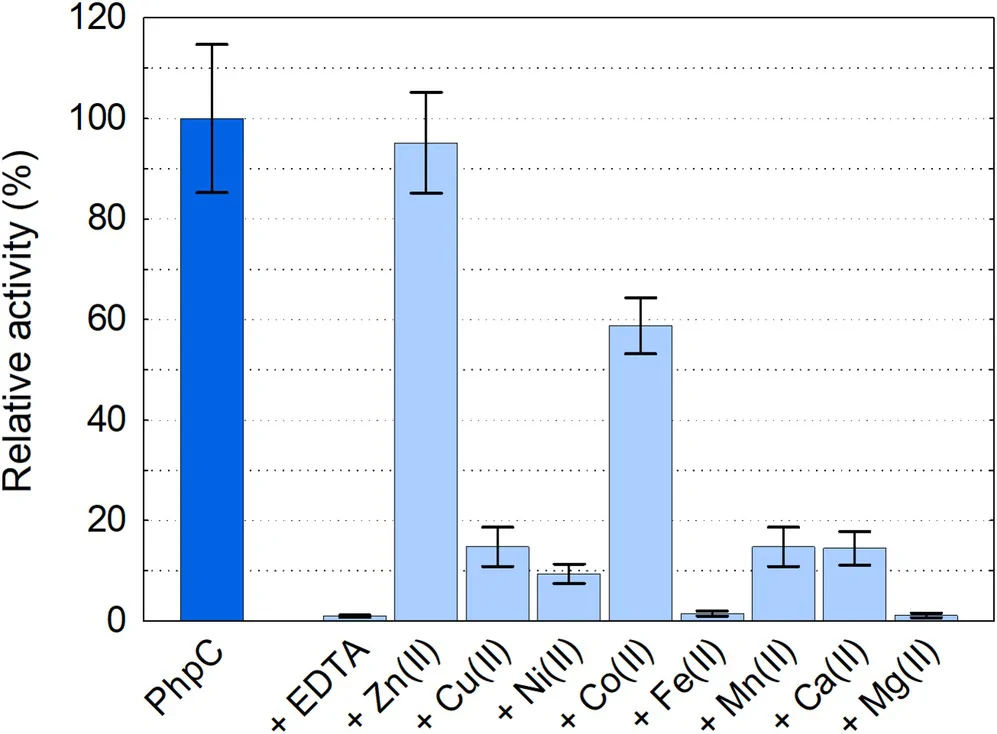
Reactivation of EDTA‐inactivated PhpC by different divalent metal ions. The dark blue bar represents the control reaction performed with the untreated enzyme. Different metals (final concentration: 50 μM) were added to the reaction mixture as chlorides.

To investigate the metal preferences of the degradative PhpC from *P. fungorum*, the enzyme was completely inactivated by extended EDTA treatment, desalted, and tested for reactivation in the presence of different metal ions, as detailed in Section 4. Relative to the untreated PhpC, we obtained a nearly full recovery of activity with Zn^2+^ and a >50% recovery also with Co^2+^; all other divalent metal ions tested activated to a much lower extent (≤15%; Figure 5).

The experiment implied a rather broad metal acceptance by PhpC, with a distinct preference for Zn^2+^ and Co^2+^. In comparison to Co^2+^, however, Zn^2+^ reactivated to a higher extent and at lower concentrations (Figure S4). Furthermore, Zn is generally much more abundant than Co in *E. coli* cells (in which the recombinant PhpC was produced) (Nies, 2007). Indeed, an ICP‐MS (inductively coupled plasma–mass spectrometry) analysis of the protein stock used for our experiments revealed the presence of Zn as the main protein‐associated metal, with significant traces of Ni and Mn, and negligible amounts of Co, Fe, and Cu. Taken together, these data indicate that PhpC is likely activated by Zn^2+^ in vivo.

### 2.6 Catalytic efficiency of PhpC and PbfG

We then established the basic catalytic parameters for the HEP dehydrogenase reactions catalyzed by PhpC and PbfG (Table 1 and Figure S5a,b).

**TABLE 1:** Apparent kinetic parameters of PhpC and PbfG in the forward and reverse reaction (50 mM TEA buffer, pH 8.0, 25°C).

Forward reaction (HEP → PAA)	*k*_cat_ (s^−1^)	*K*_*M*_^HEP^ (mM)	*k*_cat_/*K*_*M*_^HEP^ (M^−1^ s^−1^)
PhpC	0.36 ± 0.02	1.36 ± 0.3	270 ± 40
PbfG	9.9 ± 0.34	0.10 ± 0.02	88,500 ± 11,000
Reverse reaction (PAA → HEP)	*k*_cat_ (s^−1^)	*K*_*M*_^PAA^ (mM)	*k*_cat_/K_M_^PAA^ (M^−1^ s^−1^)
PhpC	3.5 ± 0.2	0.050 ± 0.010	68,000 ± 14,000
PbfG	27 ± 1.8	0.09 ± 0.02	290,000 ± 40,000

*Note*: Parameters are deemed apparent because the assays were conducted at a constant concentration of coenzyme: for the forward reaction, NAD^+^ concentration was 1.5 mM while for the reverse reaction the concentration of NADH was 0.25 mM. The reported errors were estimated by the fitting procedure.

The PhpC performance as a HEP dehydrogenase was relatively modest, with *k*_cat_ = 0.36 s^−1^ and *k*_cat_/*K*_*M*_ = 270 M^−1^ s^−1^ (Table 1). However, these parameters were in line with the properties of related pf00465 dehydrogenases: for example, sulfoacetaldehyde reductase from *B. kashiwanohense* oxidized 2‐hydroxyethanesulfonate (isethionate) with *k*_cat_ = 0.97 s^−1^ and *k*_cat_/*K*_*M*_ = 218 M^−1^ s^−1^ (Zhou et al., 2019) whereas 3‐hydroxyethylpropionate dehydrogenase from *Lysinibacillus massiliensis* oxidized its substrate with *k*_cat_ = 3.4 s^−1^ and *k*_cat_/*K*_*M*_ = 680 M^−1^ s^−1^ (Yin et al., 2019). Thus, modest catalytic efficiency appears to be a common feature of alcohol‐to‐aldehyde reactions catalyzed by this group of enzymes.

In comparison to PhpC, PbfG was a much better HEP dehydrogenase, showing a ~27 times higher turnover number (*k*_cat_ = 10 s^−1^) and a ~300‐fold higher *k*_cat_/*K*_*M*_ (88,500 M^−1^ s^−1^). The catalytic efficiency of PbfG was comparable to that of closely related NAD‐dependent dehydrogenases. As an example, threonine dehydrogenase from *E. coli* (the most similar enzyme of known function) reportedly shows *k*_cat_ = 135 s^−1^, *K*_*M*_ = 1.43 mM and *k*_cat_/*K*_*M*_ = 94,400 M^−1^ s^−1^ (Boylan & Dekker, 1981).

We also aimed at assessing the efficiency of both PhpC and PbfG in the reverse reaction (i.e., the reduction of PAA to HEP). To this end, we synthesized PAA through a new procedure (see Section 4 and Scheme S1). Unlike previously reported synthetic approaches (Gama et al., 2022; Isbell et al., 1969; Pallitsch et al., 2017), our synthetic method did not rely on bromoacetaldehyde diethylacetal, PCl_5_ and SO_2_ (all of them fatal or toxic if inhaled) or on the use of flammable hydrogen gas. Instead, PAA was prepared from diethyl methylphosphonate and dimethylformamide. Although the final yield of our procedure (18%) was lower than that of other established routes, the method is significantly safer and substantially increased the product purity to 96%, while the highest previously reported purity was 56 mol% (Pallitsch et al., 2017).

With highly pure PAA in hand, we determined the catalytic parameters for the PAA reductase reactions (Table 1 and Figure S5c,d). Notably, both PhpC and PbfG were more efficient in the reduction of PAA than in the oxidation of HEP. In particular, PhpC's performance as a PAA reductase was better than those of previously characterized biosynthetic PhpC enzymes, which showed *k*_cat_/*K*_*M*_ values in the range 1200–41,000 M^−1^ s^−1^ (and *k*_cat_ values in the 0.4 to 1.3 s^−1^ range) (Shao et al., 2008). Furthermore, although PbfG remained more efficient than PhpC also in the PAA reductase reaction, in this case the *k*_cat_/*K*_*M*_ values differed by less than fivefold between the two enzymes (Table 1).

### 2.7 Substrate specificity of PhpC and PbfG and inhibition by anionic compounds

Both PhpC and PbfG were then assayed for the ability to oxidize different potential alternative substrates: in addition to HEP, we tested AEP, isethionate, l‐threonine, ethanol, 1‐propanol, and glycolate. As shown in Figure S6, PhpC reacted appreciably only with HEP, whereas PbfG also showed some trace of activity in the presence of isethionate. The efficiency of this reaction was, however, very small, with an estimated *k*_cat_/*K*_*M*_ ≈7 M^−1^ s^−1^, four orders of magnitude lower than with HEP. Overall, these results suggested a high specificity of the two enzymes for the phosphonate substrate.

To further investigate the selectivity of the PhpC and PbfG active sites, and considering that HEP is a small anionic compound, we tested the potential inhibition of the two enzymes by some HEP analogs and by a few other negatively charged compounds. Of all the natural substances tested, only AEP showed a substantial inhibitory power (Figure S7a,b), yielding estimated *K*_*i*_ values of 4.1 ± 1.1 mM for PhpC and 0.71 ± 0.1 mM for PbfG. The common ions phosphate and sulfate, at a 15 mM concentration, had only minimal effects on the rate of the PhpC reaction, whereas for PbfG they produced an appreciable (if not impressive) decrease in activity, suggesting a greater sensitivity of the latter enzyme to anionic compounds (Figure S7a,b).

As AEP appeared to inhibit both HEP dehydrogenases (PhpC and PbfG), we tested whether, conversely, HEP could function as a competitive inhibitor towards enzymes that process AEP, namely PhnW and PbfD. Indeed, we observed a substantial decrease in catalytic activity of the transaminase PhnW from *Vibrio splendidus* and of the PbfD‐type oxidase from *Marinoblastus fucicola* in the presence of HEP (Figure S7c). The estimated inhibition constants were 2.2 ± 0.3 mM for PhnW and 0.4 ± 0.1 mM for PbfD.

### 2.8 Building structural models for PhpC and PbfG

We used AlphaFold3 (Abramson et al., 2024) to predict the structures of PhpC and PbfG, both in the absence of ligands and in the presence of NAD^+^, Zn^2+^ and the substrate HEP. High‐confidence structural models were obtained for each enzyme in monomeric and dimeric states. For both PhpC and PbfG, superposing the models of the unliganded and liganded monomer showed minimal backbone rearrangements (Figure S8), suggesting that ligand binding may occur without major conformational changes in these proteins.

**FIGURE 6:**
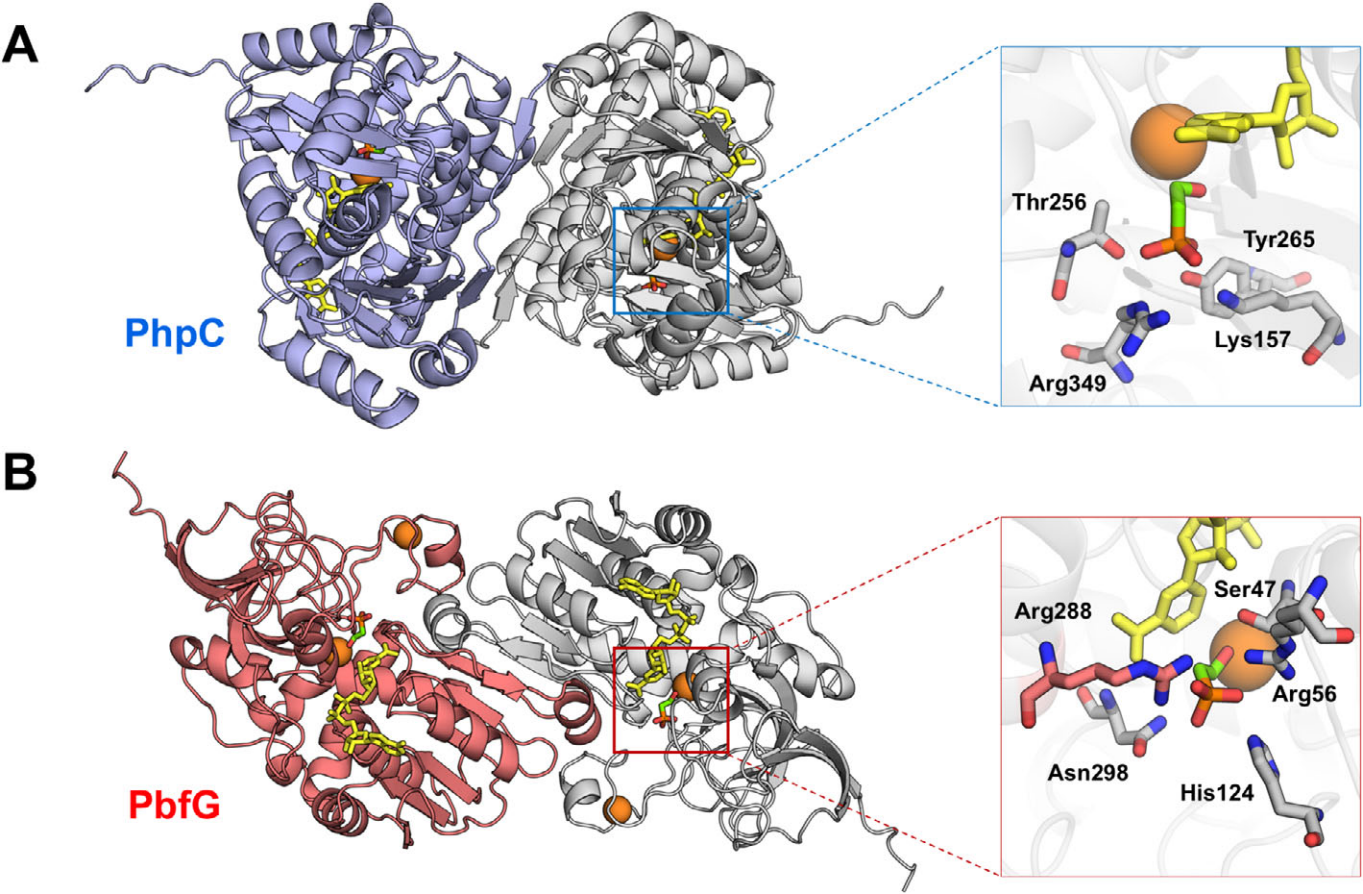
Predicted structures of the two dehydrogenases characterized in this study. In both panels, bound NAD^+^ is shown as yellow sticks, Zn^2+^ ions as orange spheres, and HEP as green sticks. (a) Model of the fully liganded PhpC dimer from *P. fungorum* predicted by AlphaFold3 (Abramson et al., 2024). The enlarged view shows the active site with bound HEP. (b) AlphaFold3‐predicted structure of the fully liganded PbfG dimer from *C. protaetiae*. The enlarged view shows the active site with bound HEP. Schematic two‐dimensional representations of the interactions formed by HEP in the active sites of PhpC and PbfG are provided in Figure S11a,b.

To assess their structural plausibility, liganded models for the monomers were aligned to the experimentally determined structures of homologous proteins: PhpC was structurally aligned to sulfoacetaldehyde reductase from *B. kashiwanohense* (PDB: 6jkp) whereas PbfG was aligned to threonine dehydrogenases from *Pyrococcus horikoshii* (PDB 2dfv) and *Thermus thermophilus* (PDB 2dq4). As detailed in Figure S9 and its caption, these checks supported a correct reproduction of the overall fold for the two HEP dehydrogenases. The dimeric assemblies were also predicted with high confidence, with ipTM/pTM values of 0.87/0.91 for the fully liganded PhpC and 0.96/0.95 for the fully liganded PbfG (Figure 6).

The PhpC structure was modeled including a single Zn^2+^ ion, consistent with the structures of related pf00465 enzymes (Ma et al., 2025; Zhou et al., 2019). Alphafold3 placed this metal ion near the active site, coordinated in a tetrahedral geometry by His274, His195, His260, and Asp191 (Figure S10a). This configuration closely matches that observed in sulfoacetaldehyde reductase, which uses His261, His275, Asp192, and Gln196 to coordinate a Zn^2+^ ion (Zhou et al., 2019). Notably, an Alphafold3 model of PhpC with Co^2+^ showed a confidence comparable to the model with Zn^2+^, and positioned the metal ion at exactly the same location, consistent with an active‐site geometry capable of accommodating different metal ions and with the results of our metal reactivation experiments (Figure 5).

The liganded structure of PbfG, in analogy with what is observed for related enzymes (Ceccarelli et al., 2004; Ishikawa et al., 2007), was modeled with two distinct Zn^2+^ ions per monomer. The first Zn^2+^ was placed by AlphaFold3 at the PbfG active site and corresponded to the catalytic zinc observed in threonine dehydrogenase. This metal occupied the same spatial position as in homologous enzymes and was coordinated by Cys45, His66, and Cys166 (Figure S10b), with the hydroxyl group of the substrate HEP completing the coordination sphere (Figure 6b). As for the second Zn^2+^ ion, AlphaFold3 placed it in a site for a structural metal, coordinated by four Cys residues (Figure S10c). The binding site and the coordination geometries were totally conserved relative to threonine dehydrogenase (Ishikawa et al., 2007).

Regarding the predicted HEP binding mode, PhpC features two positively charged residues (Lys157 and Arg349) in the substrate binding cavity. In the liganded model these residues form a salt‐bridge clamp stabilizing HEP binding, with additional interactions coming from Thr256 and above all from Tyr264 (Figures 6a and S11a). The latter residue is strictly conserved in PhpC enzymes (whereas in sulfoacetaldehyde reductases it is replaced by Phe) and was predicted to form a short H‐bond with the phosphonate group. Furthermore, in the Alphafold3 model, the C2 carbon of HEP and the reactive C4 carbon of the nicotinamide ring were only 3.3 Å apart, a spatial arrangement consistent with the expected hydride transfer between these two atoms.

As for PbfG, interaction analysis identified Arg56, His124, Asn298, and Ser47 as the key residues stabilizing substrate binding, together with coordination of the alcohol oxygen to the Zn^2+^ ion (Figures 6b and S11b). In this case, the predicted distance between C2 of HEP and C4 of the nicotinamide ring was 3.6 Å, still compatible with the expected hydride transfer between the two atoms.

## 3 DISCUSSION

The survival and proliferation of bacteria require the coordinated action of multiple genes, which are often encoded in contiguous genomic regions (clusters) that may contain all the genes necessary for a particular function. In terms of bacterial physiology and metabolism, the clustering of genes is a direct and efficient means to ensure their coexpression and coregulation (Price et al., 2005). From a bioinformatic standpoint, function prediction based on gene context can complement and enhance homology‐based approaches by leveraging the “guilt by association” principle (Aravind, 2000), according to which the function of a protein can be inferred by examining its association with other proteins whose roles are already known.

Indeed, based on an analysis of genomic context, we positively identified two groups of dehydrogenases involved in HEP catabolism. One group consists of ‘degradative’ homologs of the biosynthetic PhpC enzymes; the other is a subfamily of pf00107 dehydrogenases, which we termed PbfG. Both catalyze the oxidation of HEP to PAA, which can then be degraded by known enzymes— PhnX or the PhnY‐PhnA duo— to release inorganic phosphate.

For PhpC and PbfG, we have adopted the term “HEP dehydrogenase”. In strictly chemical terms, this designation is equivalent to “PAA reductase” (EC 1.1.1.309), the name originally assigned to the biosynthetic PhpC enzymes; however, our terminology emphasizes the catabolic role of the enzymes described here. The designation “HEP dehydrogenase” appears particularly appropriate for PbfG, as we found the *pbfG* genes consistently located within degradative gene clusters, with the only exception of two homologs identified in Actinoplanes species (Figure S2).

### 3.1 One function, two enzymes

Although PhpC and PbfG perform the same biochemical function (i.e., the NAD‐dependent interconversion of HEP and PAA), and despite the fact that both enzymes employ the coenzyme NAD^+^ and Zn^2+^ for catalysis, all the available evidence indicates that they are unrelated catalysts that have evolved independently. PhpC and PbfG are very different in terms of sequence and structure (Figure 6 a vs. b); for example, while PbfG accommodates NAD in a canonical Rossmann‐fold domain, PhpC harbors a highly divergent, non‐canonical Rossmann‐like domain. Furthermore, the two enzymes show different affinities for their metal ions (Figure 4c) and different catalytic efficiencies (Table 1).

The PhpC‐type enzymes likely emerged earlier in evolution, as suggested for example by the large divergence between the sequences of these enzymes (the average identity among the sequences in Table S1 was 39% with the two most divergent sequences sharing a 26.8% identity). Indeed, PhpC‐type enzymes may have initially evolved for biosynthetic purposes. This hypothesis is supported by the much broader taxonomic distribution of *phpC*‐containing biosynthetic clusters compared with *phpC*‐containing degradative clusters (see Figure S1 and its caption) as well as by the general rationale that the degradation of a compound necessarily presupposes its prior biosynthetic or environmental availability. At any rate, because PhpC catalyzes an inherently reversible reaction, the ancestral ‘biosynthetic’ enzymes could have been readily recruited for catabolic functions through their association with PhnX or the PhnY‐PhnA pair. Such shifts in metabolic function may have occurred independently in multiple instances during evolution (Figure S1a).

Albeit diffused in a similar number of phyla as compared to ‘degradative’ PhpC, PbfG enzymes were slightly less divergent (the average identity among the sequences in Table S2 was 40.7% with the two most diverse sequences sharing a 27.9% identity), possibly consistent with a more recent origin. PbfG sequences were very clearly separated from the closest characterized homologs, threonine dehydrogenases (Figure S2a), and *pbfG* genes were almost invariably associated with degradative clusters (Figure S2 and its caption). Based on these observations, we speculate that PbfG may have originally evolved to fulfill a catabolic role.

Remarkably, PbfG oxidized HEP with substantially greater catalytic efficiency than PhpC, showing a roughly 300‐fold greater *k*_cat_ */K*_*M*_ (Table 1). While we have no simple explanation for this gap in performance, we note two structural differences that may contribute to it. First, in the predicted liganded structure of PhpC, the hydroxyl group of HEP lies farther from the catalytic metal, whereas in PbfG it coordinates directly to Zn^2+^ (Figures 6 and S11), an interaction that may increase substrate reactivity (Plapp et al., 2017). Second, the PbfG model shows a large electropositive cleft leading into the active site (Figure S12). As HEP is negatively charged, this feature could assist catalysis by funneling the substrate towards the active site and promoting its proper orientation, analogous to mechanisms proposed for other enzymes, most notably acetylcholinesterase (Radić et al., 1997). The peculiar electrostatic surface of the *C. protaetiae* PbfG enzyme appears to be conserved in most of its homologs (Figure S13).

Considering that PbfG is a substantially more efficient HEP dehydrogenase, at least in vitro, one is left to wonder why it has not completely superseded PhpC as a degradative enzyme. One possibility is that the present‐day distribution of the two enzymes partly reflects historical contingency. If ‘degradative’ PhpC enzymes emerged earlier and in multiple instances from biosynthetic enzymes, as argued above, this may have conferred an evolutionary advantage that was difficult to overcome by PbfG. Alternatively, PhpC may offer operational advantages that PbfG lacks, such as a reduced sensitivity to inhibitors or beneficial secondary activities. Although our limited screening of potential alternative substrates did not reveal such activities (Figure S6), it is noteworthy that a PhpC homolog from *Burkholderia cenocepacia*, termed PsrA, has been implicated in the detoxication of membrane lipid peroxidation products (Naguib et al., 2022). The actual enzymatic activity of PsrA was not tested, and although its sequence is highly similar to that of PhpC from *P. fungorum*, the *psrA* gene is not genomically associated with other known genes involved in phosphonate metabolism.

### 3.2 More than ancillary enzymes

In the context of the hydrolytic pathways for AEP degradation (Figure 1a), our group recently identified a few enzymes that can convert other natural, AEP‐related aminophosphonates into PAA, which can then be processed by PhnX or by PhnY‐PhnA (Ruffolo et al., 2025; Zangelmi et al., 2021, 2023). The genes for these ancillary enzymes are typically found in clusters patently dedicated to AEP catabolism, suggesting that their purpose is to expand the usefulness of the hydrolytic AEP degradation pathways. In contrast, while PhpC and PbfG can form PAA from HEP, the corresponding genes are often found in clusters that are not dedicated to HEP degradation, as they lack both *phnW* and *pbfD* (Tables S1 and S2).

What might account for this pattern? One possibility is that some organisms, growing in particular environmental niches, may have specialized in feeding on HEP rather than on AEP. While this may be the case in certain instances, the genome of *B. fungorum* and those of many other organisms in Tables S1 and S2 (such as *Alcaligenes faecalis, Cupriavidus taiwanensis, Lysobacter gummosus, Chondinema littorale*, and *Botrimarina mediterranea*) contain separate clusters for the hydrolytic degradation of AEP. Another possibility is therefore that AEP and HEP seldom occur together in the environment, making it more convenient to have separate (and independently expressed) catabolic machineries for the two. A distinct, but not mutually exclusive, explanation is that the concomitant expression of the AEP and HEP degrading enzymes may create unwanted interferences and detours in the pathway. For example, when PAA is formed from AEP, the simultaneous presence of PhnX (which catalyzes an irreversible reaction) and PbfG (whose reaction, while reversible, is inherently shifted towards HEP formation) will initiate a tug‐of‐war over the use of PAA, ultimately slowing down its hydrolysis. An in vitro experiment aimed at illustrating the competition between the HEP dehydrogenases and PhnX is shown in Figure S14. Under physiological conditions, the inhibition exerted by AEP on PhpC and PbfG (Figure S7a,b) and, conversely, by HEP on PhnW or PbfD (Figure S7c) may further complicate the issue.

### 3.3 On the environmental abundance and biological significance of HEP

Overall, HEP degradative clusters are found in a variety of bacteria, scattered across at least eight different phyla. By inference, this suggests that HEP is a relatively abundant compound in the environment and represents a valuable source of phosphorus and energy for microorganisms. Such an environmental abundance cannot be due to the presence of HEP as an intermediate in the biosynthesis of niche secondary metabolites (Shao et al., 2008), Rather, HEP may occur in the environment primarily as a component of larger molecules, such as polysaccharides, as observed in two actinomycetes (Yu et al., 2014). Indeed, in some studies HEP has been identified as a major polysaccharide component of marine dissolved organic matter (Repeta et al., 2016; Repeta & Boiteau, 2017).

Possibly related to this, we note that several of the bacteria in Tables S1 and S2 are linked to the biodegradation of plant biomass. For example, the PhpC‐containing *P. fungorum* is often associated with the ligninolytic fungus *Phanerochaete chrysosporium* (Coenye et al., 2001). Fibrobacteria (some of which also possess degradative PhpC—Table S1) are isolated from the rumen and are involved in the degradation of plant fibers (Neumann et al., 2018). *C. protaetiae* has been isolated from the gut of the sawdust‐eating *Protaetia brevitarsis* larvae (Han et al., 2022). Planctomycetota (such as *Bremerella* sp. P1, which contains PhpC, and *Caulifigura coniformis*, which contains PbfG) are abundant in environments characterized by active biomass degradation (Klimek et al., 2025; Wang et al., 2024).

This circumstantial evidence suggests that HEP could be released during the microbial degradation of plant biomass. Free HEP has been detected in plant metabolomic studies (e.g., He et al., 2024) and we speculate that a significant fraction of this compound might occur as a component of structural polymers. Alternatively, HEP could be abundant in environments where plant fibers are degraded because other members of the microbial community in these environments produce HEP in substantial amounts.

## 4 MATERIALS AND METHODS

### 4.1 Materials

Triethanolamine (TEA), 2‐hydroxyethanesulfonate (isethionate), and 2,2‐bis(hydroxymethyl)‐2,2′,2″‐nitrilotriethanol (Bis‐Tris) were purchased from Sigma‐Aldrich (now Merck). 2‐Aminoethylphosphonate (AEP) was from Wako chemicals, l‐Threonine was from Fluka, 1‐propanol was from VWR, ethanol, and glycolic acid were from Carlo Erba, NAD^+^ was from Roche, and NADH was from Alfa Aesar. The BIOMOL Green® kit for phosphate detection was from Enzo Life Sciences.

Recombinant PhnX and PhnW enzymes from *Vibrio splendidus* and PbfD from *Mariniblastus fucicola* were produced and purified as described (Zangelmi et al., 2021, 2023). 2‐Hydroxyethylphosphonate (HEP) was synthesized as described in the literature (Hammerschmidt & Kählig, 1991; Whitteck et al., 2011).

### 4.2 Synthesis of PAA: general experimental details

NMR spectra were recorded either on a Bruker BioSpin AV NEO 400 (^1^H: 400.27 MHz, ^31^P: 162.03 MHz) or AV III HD 700 spectrometer and the recorded spectra were referenced to δ_H_ (C*H*Cl_3_) = 7.26 or δ_H_ (*H*OD) = 4.80 ppm, and δ_p_ (external H_3_PO_4_) = 0.00 ppm. Coupling constants (*J*) are given in Hz. Purities of compounds were estimated using ^31^P NMR spectra, or ^1^H NMR spectra and are given in mol%.

High resolution mass spectra (HRMS) were recorded on a Thermo Fisher Exploris 120 spectrometer using ESI ionization and an Orbitrap detector in positive mode at a resolution of ±5 ppm. Pictures of the recorded ^1^H, ^13^C and ^31^P NMR spectra of all synthesized compounds are available as Data S1.

### 4.3 Synthesis of PAA: procedure

The procedure is outlined in Scheme S1. Numbering and order of compounds is in accordance with the numbering in Scheme S1.

*Diethyl 2‐oxoethylphosphonate* (**2**): A solution of *n*BuLi (21 mmol, 5.821 g, 8.4 mL, 2.5 M in hexanes, 1.05 eq.) in 8.4 mL THF was added dropwise to a stirred solution of diethyl methylphosphonate (**1**; 20 mmol, 3.043 g, 2.92 mL, 1.0 eq.) in 4 mL THF at −78°C under argon atmosphere. After 30 min of constant stirring, the cooling bath was removed, and the reaction mixture was allowed to reach room temperature. The pH of the reaction mixture was adjusted to 1 using an aqueous 2 M HCl solution. The residue was extracted three times using DCM (3 × 30 mL). The combined organic phases were washed with brine, dried over MgSO_4_, filtered and concentrated under reduced pressure. The crude was further purified via bulb‐to‐bulb distillation (product fraction b.p. 70–120°C at 1–4 mbar), to give the product as a colorless oil (30%, 6.01 mmol, 1.082 g, 95 mol% purity); ^1^H NMR (400.27 MHz, CDCl_3_) δ_H_ 9.66 (dt, *J*_HP_ = 0.8 Hz, *J*_HH_ = 3.2, 1H, –C*H*O), 4.23–4.11 (m, 4H, 2 × –OC*H*_2_CH_3_), 3.07 (dd, *J*_HH_ = 3.2, *J*_HP_ = 21.9 Hz, 2H, –C*H*_2_–CHO), 1.34 (t, *J* = 7.1, 6H, 2 × –OCH_2_C*H*_3_) ppm; ^31^P NMR (162.03 MHz, CDCl_3_): δ_P_ 18.86 (∫ 0.95, **2**), 30.45 (∫ 0.05, unknown impurity) ppm.

*Phosphonoacetaldehyde and its hydrate as disodium salts* (**3** and **4**): Allyltrimethylsilane (11.1 mmol, 1.273 g, 1.77 mL, 2 eq.) and TMSBr (22.3 mmol, 3.411 g, 2.94 mL, 4 eq.) were added to a stirred solution of **2** in 10 mL of 1,2‐dichloroethane (DCE) under argon atmosphere. After 18 h of constant stirring, all volatiles were removed under reduced pressure, and the resulting solid was redispersed in 10 mL of DCE. The solvent was again removed; this step was repeated one more time. The residual brown solid was thoroughly dried on high vacuum before being redissolved in 10 mL of water. After 15 min of stirring, the pH of the solution was adjusted to 4.0 using a 1 M aq. NaOH solution. The product was lyophilized to give the desired phosphonic acid sodium salt **3** in equilibrium with its hydrate **4** (ratio 3.5:1) as a colorless powder (59%, 3.29 mmol, 582 mg, 96 mol% purity); HRMS calculated for C_2_H_3_Na_2_O_4_P (167.9637 g/mol): *m/z* 168.9637 [M + H]^+^, found: *m/z* 168.9637; C_2_H_5_O_4_P (124.03 g/mol): *m/z* 124.9375 [M + H]^+^, found: *m/z* 124.9375; C_2_H_4_NaO_4_P (146.01 g/mol): *m/z* 146.9818 [M + H]^+^, found: *m/z* 146.9818.

*Spectroscopical characterization of aldehyde*: ^1^H NMR (400.27 MHz, D_2_O) δ_H_ 9.62 (t, *J*_HH_ = 3.8, 1H, –C*H*O), 3.09, 3.04 (dd, *J*_HP_ = 21.2, *J*_HH_ = 3.8 Hz, –C*H*_2_CHO) ppm; ^31^P NMR (162.03 MHz, D_2_O): δ_P_ 11.2 ppm; ^13^C NMR (150.93 MHz, D_2_O): δ_C_ 201.5 (d, *J*_CP_ = 5.4 Hz, –CH_2_ *C*HO), 46.3 (d, *J*_CP_ = 120.7 Hz, –C*H*_2_CHO) ppm.

*Spectroscopical characterization of hydrate*: ^1^H NMR (400.27 MHz, D_2_O): 5.32 (dt, *J*_HP_ = 5.9, *J*_HH_ = 5.6, 1H, –CH_2_–C*H*(OH)_2_), 2.03 (dd, *J*_HP_ = 17.6, *J*_HH_ = 5.6, 2H, –C*H*_2_CH(OH)_2_) ppm; ^31^P NMR (162.03 MHz, D_2_O): δ_P_ 18.7 ppm; ^13^C NMR (176.13 MHz, D_2_O): δ_C_ 88.0 (s, –*C*H(OH)_2_), 37.1 (d, *J*_CP_ = 130.7, –C*H*_2_–CH(OH)_2_) ppm.

[Note: there is a considerable H‐D exchange between the used solvent (D_2_O) and the α‐protons next to the P‐atom in both the aldehyde and hydrate form via the respective enol‐form. This is neglectable for the recording of ^1^H NMR spectra, but needs to be taken into account for ^13^C spectra. A significant amount of deuterated **3** and **4** is already detected within the measurement time—δ_C_ = 45.99 (td, *J* = 112.5, 19.7, CD_2_‐P aldehyde) and 36.7 (td, *J* = 130.3, 18.6, CD_2_‐P hydrate)].

### 4.4 Bioinformatic analyses

Gene context was explored using tools available at the Integrated Microbial Genomes & Microbiomes (IMG/M) website (https://img.jgi.doe.gov/m/). Clusters involved in HEP degradation were initially identified through co‐occurrence of genes encoding PhpC‐ or PbfG‐type proteins together with either genes for the hydrolase PhnX or the PhnY/PhnA couple.

To identify the closest homologous enzymes of known function, the sequences of PhpC and PbfG were used for BLASTP analyses against the UniProt/Swissprot database (https://www.uniprot.org/).

The PbfG and PhpC sequences and those of their homologs were aligned with Clustal Omega (https://www.ebi.ac.uk/jdispatcher/msa/clustalo) and the sequence alignments in FASTA format were converted into Phylip interleaved format using Seqret (https://www.ebi.ac.uk/jdispatcher/sfc/emboss_seqret). To obtain a cladogram summarizing the sequence relationships according to the maximum likelihood principle, the output Phylip files were analyzed through the software tool PhyML (http://www.atgc-montpellier.fr/phyml/) with a standard bootstrap analysis of 100 repeats. The trees were then visualized by iTOL (Letunic & Bork, 2024).

To calculate percent identities between pairs of proteins, sequences were aligned with Needle (https://www.ebi.ac.uk/Tools/psa/emboss_needle/) and the number of identical positions in the alignment was divided by the length of the shorter sequence.

### 4.5 Expression and purification of recombinant HEP dehydrogenases

Synthetic genes encoding PhpC from *P. fungorum* (NCBI accession code: WP_074772540) and PbfG from *C. protaetiae* (NCBI: WP_154797266), codon‐optimized for expression in *E. coli*, were synthesized by Bio‐Fab Research (Rome, Italy). The same company also cloned *phpC* in a pET24‐C‐His‐tag vector and *pbfG* in a pET28‐N‐His‐tag vector, exploiting in both cases the NdeI/XhoI restriction sites. The plasmids were then used to transform *E. coli* Tuner (DE3) cells for expression of the His‐tagged proteins.

A preculture of transformed bacteria (10 mL) was grown overnight at 37°C and then used to inoculate 1 L of autoinducing Luria‐Bertani (LB) broth plus kanamycin (50 μg/mL), placed in a 5 L flask. Bacteria were grown at 37°C until the OD_600_ reached 0.7–0.8, then transferred at 20°C for 16 h under vigorous shaking. Eventually, the cells were harvested by centrifugation, resuspended in a suitable buffer (for PhpC: 50 mM HEPES‐Na pH 7.4, 300 mM NaCl, 2 mM β‐mercaptoethanol, 5% glycerol; for PbfG: 50 mM Tris–HCl pH 7.1, 300 mM NaCl, 2 mM β‐mercaptoethanol, 10% glycerol) supplemented with protease inhibitors (1 mM PMSF, 1 mM benzamidine) and lysed using a French press. The cell lysate was immediately centrifuged (40 min at 14000 g rcf, 4°C), the supernatants were loaded onto a HisTrap™ Fast Flow column (Cytiva) and the recombinant proteins were purified with an ÅKTA Pure FPLC System. Protein purity in the eluted fractions was assessed by SDS‐PAGE and fractions with purity >95% were pooled and dialyzed against a storage buffer containing 50 mM HEPES‐Na pH 7.4, 300 mM NaCl, 2 mM β‐mercaptoethanol, 5% glycerol for PhpC and 50 mM Tris–HCl pH 7.1, 300 mM NaCl, 2 mM β‐mercaptoethanol, 10% glycerol for PbfG.

The protein concentration was estimated based on extinction coefficients (ε_280_ = 36,440 M^−1^ cm^−1^ for PhpC and 24,980 M^−1^ cm^−1^ for PbfG) calculated with ProtParam (http://web.expasy.org/protparam/). The final yields for PhpC and PbfG were, respectively, 13.4 and 2.9 mg of purified protein per liter of bacterial culture. The stocks of purified proteins were stored at −80°C in 0.5 mL aliquots.

### 4.6 Evaluation of the PhpC and PbfG oligomerization state

The oligomerization state of the purified enzymes was assessed by analytical size‐exclusion chromatography. A 60‐μL aliquot of either PhpC (70 μM, monomer concentration) or PbfG (36 μM) was run through a Superdex® 200 Increase 5/150 GL column (Cytiva) on an ÅKTA® Pure System FPLC (GE Healthcare). For both enzymes, the mobile phase was a 25 mM Tris–HCl buffer (pH 7.0) containing 150 mM NaCl. The flow rate was 0.3 mL/min. A calibration chromatogram was recorded on the same day and under the same conditions using a set of proteins of known molecular mass.

### 4.7 Kinetic assays of PhpC and PbfG activity

The oxidation of HEP to PAA with NAD^+^ or NADP^+^ as the electron acceptor was monitored spectrophotometrically, by following the rise in absorption at 340 nm associated with the accumulation of NAD(P)H. The amount of NAD(P)H formed was quantitated based on ε_340nm_ = 6220 M^−1^ cm^−1^ for the reduced coenzymes. The reactions were carried out at 25°C in a 150 μL final volume; each reaction contained 50 mM TEA‐HCl pH 8, 1.5 mM NAD^+^ (or NADP^+^), along with HEP and the enzyme being investigated. Measurements were conducted using a thermostated Jasco V‐750 UV–Vis spectrophotometer.

To obtain the kinetic parameters for the two dehydrogenases, reactions at different substrate concentrations were repeated in triplicate and the observed rates were nonlinearly least‐squares fitted to Equation (1).

(1) $$\frac{v_{0}}{\left[E\right]}=\frac{k_{\mathrm{cat}}\times {\left[S\right]}_{0}}{K_{M}+{\left[S\right]}_{0}}$$

where *v*_0_ is the observed initial rate (in μM/s), [*E*] is the enzyme concentration (in μM), [*S*]_0_ is the initial concentration of substrate, *k*_cat_ is the turnover number (in s^−1^), and *K*_*M*_ is the Michaelis–Menten constant. Data were fitted with SigmaPlot v14.5 (Systat Software Inc.).

### 4.8 Phosphate release assay

Phosphate release was assessed using the BIOMOL® Green kit (Enzo Life Sciences) in microtiter format. The reaction with PhpC contained 50 mM TEA‐HCl buffer pH 8, the enzyme under examination, 1 μM PhnX, 1 mM MgCl_2_, 1.5 mM NAD^+^, 1 mM β‐mercaptoethanol, and the substrate. For PbfG, the reaction mixture was the same, but 0.1% bovine serum albumin (BSA; Thermo Scientific Chemicals) was added to stabilize the enzyme. Reactions were incubated for 4 h at room temperature, then 15 μL of each mixture was transferred into 200 μL of BIOMOL® Green reagent to block the enzyme activity. Color development was evaluated after 30 min.

### 4.9 Reactivation of M^2^ ^+^‐depleted PhpC

The preferences of PhpC from *P. fungorum* towards different divalent metal ion cofactors were tested based on a procedure previously used for a biosynthetic PhpC (Shao et al., 2008). To remove the metal(s) bound to the native enzyme, a PhpC stock (25 μM) was incubated for 2 h in the presence of 25 mM EDTA, after which the enzyme activity was completely abolished. The PhpC solution was then loaded onto a PD SpinTrap™ G‐25 desalting column to remove free EDTA and the EDTA‐metal complexes.

Reactivation of the EDTA‐treated PhpC was then assessed by testing the enzyme activity in solutions that contained metal ions. After trying different protocols, the most reproducible results (and the highest degrees of reactivation) were obtained by adding the EDTA‐treated enzyme last, to a reaction mixture that contained the substrates (5 mM HEP and 1.5 mM NAD^+^) and various metal chlorides (when iron was the metal to be tested, the FeCl_2_ stock solution was prepared freshly in the presence of an excess ascorbate, to prevent metal oxidation). The buffer was 50 mM TEA and 20 mM Bis‐Tris, pH 8.0. The presence of Bis‐Tris was meant to help solubilize the transition metal ions (Liu & Sen, 2010); however, in some cases reactivation was also tested omitting Bis‐Tris, yielding essentially identical results.

### 4.10 Inductively coupled plasma–mass spectrometry (ICP‐MS)

Mn, Fe, Co, Ni, Cu, and Zn in a sample of native PhpC were determined by ICP‐MS using an Agilent 8900 triple quadrupole spectrometer at Department Geosciences, University of Padua (Italy). Instrumental calibration was carried out using certified solutions, and a known amount of Re and Rh was also introduced in each sample as an internal standard. Accuracy and precision were determined using several international reference standards, being lower than 10% of the measured value.

### 4.11 Inhibition assays

The activities of recombinant PhpC and PbfG, as well as those of PhnW from *Vibrio splendidus* (Zangelmi et al., 2021) and PbfD from *Mariniblastus fucicola* (Zangelmi et al., 2023) were measured in the presence of different compounds that could serve as competitive inhibitors. The reactions of PhpC and PbfG with HEP were monitored as described above; whereas the reactions of PhnW and PbfD with AEP were monitored through a coupled assay with PhnX and yeast alcohol dehydrogenase, as reported previously (Zangelmi et al., 2021, 2023).

When testing potential inhibitors, the inhibition constant *K*_*i*_ was estimated by fitting the data to Equation (2), which assumes a purely competitive inhibition pattern (Schiroli et al., 2013):

(2) $$\frac{v_{i}}{v_{0}}=\frac{K_{i}\left(K_{M}+{\left[S\right]}_{0}\right)}{K_{i}\left(K_{M}+{\left[S\right]}_{0}\right)+K_{M}\left[I\right]}$$

where *v*_*i*_ is the observed reaction rate, *v*_0_ is the rate in the absence of inhibitor, [*I*] is the concentration of inhibitor, [*S*]_0_ is the concentration of substrate, and *K*_*M*_ is the Michaelis–Menten constant for HEP or AEP.

### 4.12 Structural modeling of the PhpC and PbfG enzymes

Structural models of PhpC and PbfG were predicted using AlphaFold3 (Abramson et al., 2024) installed locally and executed on NVIDIA A100 80GB GPUs. Predictions were performed for unliganded monomeric and dimeric proteins, as well as for ligand‐bound complexes that included NAD^+^, divalent metal ions (Co^2+^ or Zn^2+^), and HEP (PubChem CID 89954). The SMILES representation of HEP was protonated at pH 7.4 using Open Babel (3.1.1) before model generation. Model confidence metrics (pTM and ipTM) were extracted from AlphaFold3 best predicted model.

Structural similarity searches were conducted using the Foldseek webserver with default parameters. RMSD values were calculated on Cα atoms using the align command in PyMOL 3. Electrostatic surface potentials were calculated using APBS electrostatics, implemented through the PyMOL 3 plugin.

## AUTHOR CONTRIBUTIONS

**Francesca Piazza:** Visualization; writing – review and editing; investigation; formal analysis; methodology. **Florian Götzinger:** Methodology; investigation; writing – review and editing; resources. **Katharina Pallitsch:** Methodology; resources; funding acquisition; writing – review and editing; supervision. **Alessio Peracchi:** Conceptualization; formal analysis; supervision; funding acquisition; writing – original draft; project administration. **Claudio Rivetti:** Formal analysis; supervision; visualization; writing – review and editing. **Giovanni Merici:** Visualization; software; data curation; writing – review and editing. **Marianna Rodella:** Data curation; investigation; writing – review and editing. **Nicholas Salanti:** Investigation; validation; writing – review and editing. **Elisa Rossi:** Data curation; investigation; writing – review and editing.

## CONFLICT OF INTEREST STATEMENT

The authors declare no competing interests with regard to the content of this study.

## Supporting information


**TABLE S1.** Representative PhpC sequences and composition of the corresponding degradative gene clusters.
**TABLE S2.** Representative PbfG sequences and composition of the corresponding degradative gene clusters.
**FIGURE S1.** Circular cladogram for PhpC‐type enzymes and biosynthetic gene clusters with *phpC*.
**FIGURE S2.** Circular cladogram for PbfG‐type enzymes and biosynthetic gene clusters with *pbfG*.
**FIGURE S3.** Determination of the oligomeric state of recombinant PhpC and PbfG by size‐exclusion chromatography.
**FIGURE S4.** Reactivation of EDTA‐treated PhpC by Zn^2+^ and Co^2+^.
**FIGURE S5.** Michaelis–Menten plots of the PhpC and PbfG reactions.
**FIGURE S6.** Activity of PhpC and PbfG towards HEP analogs.
**FIGURE S7.** Inhibition assays with PhpC, PbfG, PhnW, and PbfD.
**FIGURE S8.** AF3 models of ligand‐bound and ligand‐free PhpC and PbfG monomers.
**FIGURE S9.** Comparison of AF3 models of PhpC and PbfG with the X‐ray structures of Homologous proteins.
**FIGURE S10.** Predicted Zn^2+^ binding sites in the PhpC and PbfG models.
**FIGURE S11.** Interactions of HEP within the active sites of PhpC and PbfG.
**FIGURE S12.** Comparison of the electrostatic surface potentials of PhpC and PbfG.
**FIGURE S13.** Conservation of the positively charged cleft in PbfG enzymes.
**FIGURE S14.** Competition between the HEP dehydrogenases and PhnX for PAA.
**SCHEME S1.** Chemical synthesis of PAA.
**DATA S1.** NMR spectra of all the compounds chemically synthesized for the preparation of PAA.

## Data Availability

The data that support the findings of this study are available from the corresponding author upon reasonable request.

## References

[pro70783-bib-0001] Abramson J , Adler J , Dunger J , Evans R , Green T , Pritzel A , et al. Accurate structure prediction of biomolecular interactions with AlphaFold 3. Nature. 2024;630(8016):493–500. 38718835 10.1038/s41586-024-07487-w PMC11168924

[pro70783-bib-0002] Aravind L . Guilt by association: contextual information in genome analysis. Genome Res. 2000;10:1074–1077. 10958625 10.1101/gr.10.8.1074

[pro70783-bib-0003] Aronson BD , Somervilles RL , Epperly BR , Dekker EE . The primary structure of *Escherichia coli* L‐threonine dehydrogenase. J Biol Chem. 1989;264(9):5226–5232. 2647748

[pro70783-bib-0004] Blodgett JAV , Thomas PM , Li G , Velasquez JE , van der Donk WA , Kelleher NL , et al. Unusual transformations in the biosynthesis of the antibiotic phosphinothricin tripeptide. Nat Chem Biol. 2007;3(8):480–485. 17632514 10.1038/nchembio.2007.9 PMC4313788

[pro70783-bib-0005] Born DA , Ulrich EC , Ju K‐S , Peck SC , van der Donk WA , Drennan CL . Structural basis for methylphosphonate biosynthesis. Science. 2017;358(6368):1336–1339. 29217579 10.1126/science.aao3435 PMC5901744

[pro70783-bib-0006] Boylan SA , Dekker EE . L‐threonine dehydrogenase. Purification and properties of the homogeneous enzyme from *Escherichia coli* K‐12. J Biol Chem. 1981;256(4):1809–1815. 6780553

[pro70783-bib-0007] Ceccarelli C , Liang Z‐X , Strickler M , Prehna G , Goldstein BM , Klinman JP , et al. Crystal structure and amide H/D exchange of binary complexes of alcohol dehydrogenase from *Bacillus stearothermophilus*: insight into thermostability and cofactor binding. Biochemistry. 2004;43(18):5266–5277. 15122892 10.1021/bi049736p

[pro70783-bib-0008] Chin JP , McGrath JW , Quinn JP . Microbial transformations in phosphonate biosynthesis and catabolism, and their importance in nutrient cycling. Curr Opin Chem Biol. 2016;31:50–57. 26836350 10.1016/j.cbpa.2016.01.010

[pro70783-bib-0009] Coenye T , Laevens S , Willems A , Ohle M , Hannant W , Govan J , et al. *Burkholderia fungorum* sp. nov. and *Burkholderia caledonica* sp. nov., two new species isolated from the environment, animals and human clinical samples. Int J Syst Evol Microbiol. 2001;51:1099–1107. 11411678 10.1099/00207713-51-3-1099

[pro70783-bib-0010] Dumora C , Lacoste A‐M , Cassaigne A . Phosphonoacetaldehyde hydrolase from *pseudomonas aeuginosa*: purification properties and comparison with *Bacillus cereus* enzyme. Biochim Biophys Acta. 1989;997(3):193–198. 2504289 10.1016/0167-4838(89)90186-6

[pro70783-bib-0011] Elleuche S , Fodor K , von der Heyde A , Klippel B , Wilmanns M , Antranikian G . Group III alcohol dehydrogenase from *Pectobacterium atrosepticum*: insights into enzymatic activity and organization of the metal ion‐containing region. Appl Microbiol Biotechnol. 2014;98(9):4041–4051. 24265029 10.1007/s00253-013-5374-z

[pro70783-bib-0012] Fox EM , Mendz GL . Phosphonate degradation in microorganisms. Enzyme Microb Technol. 2006;40(1):145–150.

[pro70783-bib-0013] Furtak A , Szafranek‐Nakonieczna A , Furtak K , Pytlak A . A review of organophosphonates, their natural and anthropogenic sources, environmental fate and impact on microbial greenhouse gases emissions: identifying knowledge gaps. J Environ Manage. 2024;355:120453. 38430886 10.1016/j.jenvman.2024.120453

[pro70783-bib-0014] Gama SR , Stankovic T , Hupp K , al Hejami A , McClean M , Evans A , et al. Biosynthesis of the fungal organophosphonate fosfonochlorin involves an iron(II) and 2‐(oxo)glutarate dependent oxacyclase. Chembiochem. 2022;23(2):e202100352. 34375042 10.1002/cbic.202100352

[pro70783-bib-0015] Hammerschmidt F , Kählig H . Biosynthesis of natural products with a P‐C bond. 7. Synthesis of [1,1‐^2^H_2_]‐, [2,2‐^2^H_2_]‐, (*R*)‐ and (*S*)‐[1‐^2^H_1_](2‐hydroxyethyl) phosphonic acid and (*R,S*)‐[1‐^2^H_1_](1,2‐dihydroxyethyl) phosphonic acid and incorporation studies into fosfomycin in *Streptomyces fradiae* . J Org Chem. 1991;56:2364–2370.

[pro70783-bib-0016] Han H , Nguyen TTH , Li Z , Shin NR , Kim SG . *Cellulosimicrobium protaetiae* sp. nov., isolated from the gut of the larva of *Protaetia brevitarsis seulensis* . Int J Syst Evol Microbiol. 2022;72(3):005296. 10.1099/ijsem.0.005296 35348452

[pro70783-bib-0017] He T , Chen L , Wu Y , Wang J , Wu Q , Sun J , et al. Combined metabolome and transcriptome analyses of maize leaves reveal global effect of biochar on mechanisms involved in anti‐herbivory to *Spodoptera frugiperda* . Metabolites. 2024;14(9):498. 39330505 10.3390/metabo14090498 PMC11433984

[pro70783-bib-0018] Horsman GP , Zechel DL . Phosphonate biochemistry. Chem Rev. 2017;117(8):5704–5783. 27787975 10.1021/acs.chemrev.6b00536

[pro70783-bib-0019] Isbell AF , Englert LF , Rosenberg H . Phosphonoacetaldehyde. J Org Chem. 1969;34(3):755–756.

[pro70783-bib-0020] Ishikawa K , Higashi N , Nakamura T , Matsuura T , Nakagawa A . The first crystal structure of L‐threonine dehydrogenase. J Mol Biol. 2007;366(3):857–867. 17188300 10.1016/j.jmb.2006.11.060

[pro70783-bib-0021] Kafarski P . Phosphonates: their natural occurrence and physiological role. In: Churchill DG , Sikirić MD , Čolović B , Milhofer HF , editors. Contemporary topics about phosphorus in biology and materials. Volume 1. London: Intech Open; 2019. p. 1–19.

[pro70783-bib-0022] Kaya K , Morrison LF , Codd GA , Metcalf JS , Sano T , Takagi H , et al. A novel biosurfactant, 2‐acyloxyethylphosphonate, isolated from waterblooms of *Aphanizomenon flos‐aquae* . Molecules. 2006;11:539–548. 17971725 10.3390/11070539 PMC6148651

[pro70783-bib-0023] Kim A , Benning MM , OkLee S , Quinn J , Martin BM , Holden HM , et al. Divergence of chemical function in the alkaline phosphatase superfamily: structure and mechanism of the P‐C bond cleaving enzyme phosphonoacetate hydrolase. Biochemistry. 2011;50(17):3481–3494. 21366328 10.1021/bi200165h PMC3102601

[pro70783-bib-0024] Kim H , Chin J , Choi H , Baek K , Lee TG , Park SE , et al. Phosphoiodyns A and B, unique phosphorus‐containing iodinated polyacetylenes from a Korean sponge *Placospongia* sp. Org Lett. 2013;15(1):100–103. 23240782 10.1021/ol3031318

[pro70783-bib-0025] Klimek D , Lage OM , Calusinska M . Phylogenetic diversity and community structure of Planctomycetota from plant biomass‐rich environments. Front Microbiol. 2025;16:1579219. 40510668 10.3389/fmicb.2025.1579219 PMC12159837

[pro70783-bib-0026] Lantz SR , Casida JE . Characterization of the transient oxaphosphetane BChE inhibitor formed from spontaneously activated ethephon. Chem Res Toxicol. 2013;26(9):1320–1322. 23927580 10.1021/tx4002429 PMC3775443

[pro70783-bib-0027] Letunic I , Bork P . Interactive tree of life (iTOL) v6: recent updates to the phylogenetic tree display and annotation tool. Nucleic Acids Res. 2024;52(W1):W78–W82. 38613393 10.1093/nar/gkae268 PMC11223838

[pro70783-bib-0028] Li S , Horsman GP . An inventory of early branch points in microbial phosphonate biosynthesis. Microb Genom. 2022;8(2):000781. 35188456 10.1099/mgen.0.000781 PMC8942020

[pro70783-bib-0029] Liu Y , Sen D . Local rather than global folding enables the lead‐dependent activity of the 8‐17 deoxyribozyme: evidence from contact photo‐crosslinking. J Mol Biol. 2010;395(2):234–241. 19917290 10.1016/j.jmb.2009.11.028

[pro70783-bib-0030] Lockwood S , Greening C , Baltar F , Morales SE . Global and seasonal variation of marine phosphonate metabolism. ISME J. 2022;16(9):2198–2212. 35739297 10.1038/s41396-022-01266-z PMC9381506

[pro70783-bib-0031] Ma B‐D , Li J‐Y , Xu J‐H , Yu T , Kong X‐D . ADP‐ribose is a competitive inhibitor of methanol dehydrogenases from *Bacillus methanolicus* . J Biol Chem. 2025;301(9):110599. 40818608 10.1016/j.jbc.2025.110599 PMC12926040

[pro70783-bib-0032] Manav MC , Sofos N , Hove‐Jensen B , Brodersen DE . The Abc of phosphonate breakdown: a mechanism for bacterial survival. Bioessays. 2018;40(11):e1800091. 30198068 10.1002/bies.201800091

[pro70783-bib-0033] Martinez A , Tyson GW , DeLong EF . Widespread known and novel phosphonate utilization pathways in marine bacteria revealed by functional screening and metagenomic analyses. Environ Microbiol. 2010;12(1):222–238. 19788654 10.1111/j.1462-2920.2009.02062.x

[pro70783-bib-0034] McGrath JW , Wisdom GB , McMullan G , Larkin MJ , Quinn JP . The purification and properties of phosphonoacetate hydrolase, a novel carbon‐phosphorus bond‐cleavage enzyme from *Pseudomonas fluorescens* 23F. Eur J Biochem. 1995;234(1):225–230. 8529644 10.1111/j.1432-1033.1995.225_c.x

[pro70783-bib-0035] Metcalf WW , Griffin BM , Cicchillo RM , Gao J , Janga SC , Cooke HA , et al. Synthesis of methylphosphonic acid by marine microbes: a source for methane in the aerobic ocean. Science (1979). 2012;337(6098):1104–1107. 10.1126/science.1219875 PMC3466329 22936780

[pro70783-bib-0036] Naguib M , Feldman N , Zarodkiewicz P , Shropshire H , Biamis C , El‐Halfawy OM , et al. An evolutionary conserved detoxification system for membrane lipid–derived peroxyl radicals in gram‐negative bacteria. PLoS Biol. 2022;20(5):e3001610. 35580139 10.1371/journal.pbio.3001610 PMC9113575

[pro70783-bib-0037] Neumann AP , Suen G , Rodrigues JM . The phylogenomic diversity of herbivore‐associated *Fibrobacter* spp. is correlated to lignocellulose‐degrading potential. mSphere. 2018;3(6):e00593‐18. 30541780 10.1128/mSphere.00593-18 PMC6291624

[pro70783-bib-0038] Nies DH . Bacterial transition metal homeostasis. In: Nies, DH ., Silver, S . (eds) Molecular Microbiology of Heavy Metals. Microbiology Monographs, Springer, Berlin, Heidelberg. vol. 6, 2007. p. 117–142.

[pro70783-bib-0039] Pallitsch K , Rogers MP , Andrews FH , Hammerschmidt F , McLeish MJ . Phosphonodifluoropyruvate is a mechanism‐based inhibitor of phosphonopyruvate decarboxylase from *Bacteroides fragilis* . Bioorg Med Chem. 2017;25(16):4368–4374. 28693916 10.1016/j.bmc.2017.06.013

[pro70783-bib-0040] Pallitsch K , Zechel DL . The functional importance of bacterial oxidative phosphonate pathways. Biochem Soc Trans. 2023; 51(2):487–499. 36892197 10.1042/BST20220479

[pro70783-bib-0041] Plapp BV , Savarimuthu BR , Ferraro DJ , Rubach JK , Brown EN , Ramaswamy S . Horse liver alcohol dehydrogenase: zinc coordination and catalysis. Biochemistry. 2017;56(28):3632–3646. 28640600 10.1021/acs.biochem.7b00446 PMC5518280

[pro70783-bib-0042] Price MN , Huang KH , Arkin AP , Alm EJ . Operon formation is driven by co‐regulation and not by horizontal gene transfer. Genome Res. 2005;15(6):809–819. 15930492 10.1101/gr.3368805 PMC1142471

[pro70783-bib-0043] Qi X , Yun J , Qi Y , Zhang H , Wang F , Guo Q , et al. Expression and characterization of a novel 1,3‐propanediol dehydrogenase from *lactobacillus brevis* . Appl Biochem Biotechnol. 2016;179(6):959–972. 26961082 10.1007/s12010-016-2043-6

[pro70783-bib-0044] Radić Z , Kirchhoff PD , Quinn DM , McCammon JA , Taylor P . Electrostatic influence on the kinetics of ligand binding to acetylcholinesterase. J Biol Chem. 1997;272(37):23265–23277. 9287336 10.1074/jbc.272.37.23265

[pro70783-bib-0045] Repeta DJ , Boiteau RM . Organic nutrient chemistry and the marine microbiome. In: The chemistry of microbiomes: Proceedings of a seminar series. Washington, DC: National Academies Press. 2017. p. 43–52. 28806041

[pro70783-bib-0046] Repeta DJ , Ferrón S , Sosa OA , Johnson CG , Repeta LD , Acker M , et al. Marine methane paradox explained by bacterial degradation of dissolved organic matter. Nat Geosci. 2016;9(12):884–887.

[pro70783-bib-0047] Ruffolo F , Conciatori S , Merici G , Dinhof T , Chin JP , Rivetti C , et al. Genomic context analysis enables the discovery of an unusual NAD‐dependent racemase in phosphonate catabolism. FEBS J. 2025;292:4272–4288. 40384479 10.1111/febs.70130 PMC12366243

[pro70783-bib-0048] Ruffolo F , Dinhof T , Murray L , Zangelmi E , Chin JP , Pallitsch K , et al. The microbial degradation of natural and anthropogenic phosphonates. Molecules. 2023;28(19):6863. 37836707 10.3390/molecules28196863 PMC10574752

[pro70783-bib-0049] Schiroli D , Cirrincione S , Donini S , Peracchi A . Strict reaction and substrate specificity of AGXT2L1, the human *O*‐phosphoethanolamine phospho‐lyase. IUBMB Life. 2013;65(7):645–650. 23761375 10.1002/iub.1178

[pro70783-bib-0050] Shao Z , Blodgett JAV , Circello BT , Eliot AC , Woodyer R , Li G , et al. Biosynthesis of 2‐hydroxyethylphosphonate, an unexpected intermediate common to multiple phosphonate biosynthetic pathways. J Biol Chem. 2008;283(34):23161–23168. 18544530 10.1074/jbc.M801788200 PMC2516978

[pro70783-bib-0051] Sosa OA , Repeta DJ , DeLong EF , Ashkezari MD , Karl DM . Phosphate‐limited ocean regions select for bacterial populations enriched in the carbon–phosphorus lyase pathway for phosphonate degradation. Environ Microbiol. 2019;21(7):2402–2414. 30972938 10.1111/1462-2920.14628 PMC6852614

[pro70783-bib-0052] Wang JX , Wang J , Liu JQ , Li J , Jiang WX , Xu F , et al. The complete genome sequence of the planctomycetotal bacterium *Bremerella* sp. P1 with abundant genes involved in polysaccharide degradation. Mar Genomics. 2024;76:101126. 39009497 10.1016/j.margen.2024.101126

[pro70783-bib-0053] Whitteck JT , Malova P , Peck SC , Cicchillo RM , Hammerschmidt F , Van Der Donk WA . On the stereochemistry of 2‐hydroxyethylphosphonate dioxygenase. J Am Chem Soc. 2011;133(12):4236–4239. 21381767 10.1021/ja1113326 PMC3069692

[pro70783-bib-0054] Yin J , Wei Y , Liu D , Hu Y , Lu Q , Ang EL , et al. An extended bacterial reductive pyrimidine degradation pathway that enables nitrogen release from β‐alanine. J Biol Chem. 2019;294(43):15662–15671. 31455636 10.1074/jbc.RA119.010406 PMC6816092

[pro70783-bib-0055] Yu X , Price NPJ , Evans BS , Metcalf WW . Purification and characterization of phosphonoglycans from *Glycomyces* sp. strain NRRL B‐16210 and *Stackebrandtia nassauensis* NRRL B‐16338. J Bacteriol. 2014;196(9):1768–1779. 24584498 10.1128/JB.00036-14 PMC3993330

[pro70783-bib-0056] Zangelmi E , Ruffolo F , Dinhof T , Gerdol M , Malatesta M , Chin JP , et al. Deciphering the role of recurrent FAD‐dependent enzymes in bacterial phosphonate catabolism. IScience. 2023;26:108108. 37876809 10.1016/j.isci.2023.108108 PMC10590968

[pro70783-bib-0057] Zangelmi E , Stanković T , Malatesta M , Acquotti D , Pallitsch K , Peracchi A . Discovery of a new, recurrent enzyme in bacterial phosphonate degradation: (*R*)‐1‐hydroxy‐2‐aminoethylphosphonate ammonia‐lyase. Biochemistry. 2021;60(15):1214–1225. 33830741 10.1021/acs.biochem.1c00092 PMC8154272

[pro70783-bib-0058] Zhang Y , Pham TM , Kayrouz C , Ju KS . Biosynthesis of argolaphos illuminates the unusual biochemical origins of aminomethylphosphonate and Nϵ‐hydroxyarginine containing natural products. J Am Chem Soc. 2022;144(22):9634–9644. 35616638 10.1021/jacs.2c00627 PMC9284158

[pro70783-bib-0059] Zhou Y , Wei Y , Nanjaraj Urs AN , Lin L , Xu T , Hu Y , et al. Identification and characterization of a new sulfoacetaldehyde reductase from the human gut bacterium *Bifidobacterium kashiwanohense* . Biosci Rep. 2019;39(6):BSR20190715. 31123167 10.1042/BSR20190715 PMC6616044

