# Supplementary material for: Two distinct alcohol dehydrogenase classes mediate bacterial 2‐hydroxyethylphosphonate catabolism
Source: Protein Sci. 2026 Sep 15;35(10):e70783. doi: 10.1002/pro.70783 (PMC13576164; doi:10.1002/pro.70783)
Supplement: Supplementary file 1 [file PRO-35-e70783-s001.pdf]

Supporting Information to:

Two distinct alcohol dehydrogenase classes mediate bacterial 2-hydroxyethylphosphonate catabolism

Francesca Piazza[1], Marianna Rodella[1], Nicholas Salanti[1], Giovanni Merici[1], Elisa Rossi[1], Claudio Rivetti[1], Florian Götzinger[2], Katharina Pallitsch[2], Alessio Peracchi[1‡]

[1] Department of Chemistry, Life Sciences and Environmental Sustainability, University of Parma, I-43124 Parma, Italy.

[2] Institute of Organic Chemistry, University of Vienna, Währingerstrasse 38, A-1090 Vienna, Austria

LIST OF CONTENTS

		Page
**Supplementary tables**		
Table S1	Representative PhpC sequences and composition of the corresponding degradative gene clusters.	3
Table S2	Representative PbfG sequences and composition of the corresponding degradative gene clusters.	4
**Supplementary figures**		
Figure S1	Circular cladogram for PhpC-type enzymes and biosynthetic gene clusters with *phpC*	5
Figure S2	Circular cladogram for PbfG-type enzymes and biosynthetic gene clusters with *pbfG*	7
Figure S3	Determination of the oligomeric state of recombinant PhpC and PbfG by size-exclusion chromatography	9
Figure S4	Reactivation of EDTA-treated PhpC by Zn^{2+} and Co^{2+}	10
Figure S5	Michaelis-Menten plots of the PhpC and PbfG reactions	11
Figure S6	Activity of PhpC and PbfG toward HEP analogs	12
Figure S7	Inhibition assays with PhpC, PbfG, PhnW, and PbfD	13
Figure S8	AF3 models of ligand-bound and ligand-free PhpC and PbfG monomers	14
Figure S9	Comparison of AF3 models of PhpC and PbfG with the X-ray structures of homologous proteins	15
Figure S10	Predicted Zn^{2+} binding sites in the PhpC and PbfG models	17
Figure S11	Interactions of HEP within the active sites of PhpC and PbfG	18
Figure S12	Comparison of the electrostatic surface potentials of PhpC and PbfG	19
Figure S13	Conservation of the positively charged cleft in PbfG enzymes	20
Figure S14	Competition between the HEP dehydrogenases and PhnX for PAA	21
**Supplementary schemes**		
Scheme S1	Chemical synthesis of PAA	22
**Supplementary data**		
Data S1	NMR spectra of all the compounds chemically synthesized for the preparation of PAA	23
**Supporting information references**		29

Table S1 - Representative PhpC sequences and composition of the corresponding degradative gene clusters. The proteins belong to pf00465. All the gene products in this Table are present both in the NCBI and in the IMG/MER databases. A cladogram representing the relationships between these sequences is provided in Figure S1A.

**Organism**	**Order**	**NCBI Accession ID**	**Other genes in cluster**				
**Pseudomonadota**			*phnW*	*pbfD*	*phnX*	*phnY*	*phnA*
Paraburkholderia fungorum	Burkholderiales	WP_074772540			+		
Burkholderia plantarii	Burkholderiales	WP_055140978			+		
Ottowia thiooxydans	Burkholderiales	WP_028605610				+	+
Roseateles amylovorans	Burkholderiales	WP_261755781		+		+	+
Alcaligenes faecalis	Burkholderiales	WP_086061338			+		
Lampropedia aestuarii	Burkholderiales	WP_136405402		+	+		
Delftia acidovorans	Burkholderiales	WP_012204331			+		
Cupriavidus taiwanensis	Burkholderiales	WP_116380058			+		
Collimonas pratensis	Burkholderiales	WP_061943604				+	+
Herbaspirillum huttiense	Burkholderiales	WP_134222609			+		
Microbulbifer sp. ALW1	Burkholderiales	WP_160154203			+		
Silvimonas soli	Neisseriales	WP_283147580			+		
Usitatibacter palustris	Nitrosomonadales	WP_212758232				+	+
Bosea lathiri	Hyphomicrobiales	WP_103874160				+	+
Bradyrhizobium elkanii	Hyphomicrobiales	WP_018272036				+	+
Hansschlegelia beijingensis	Hyphomicrobiales	WP_183396101				+	+
Lysobacter gummosus	Lysobacterales	WP_057942983				+	+
Pseudomonas putida GB-1	Pseudomonadales	ABY99150			+		
Pseudomonas abietaniphila	Pseudomonadales	WP_074754111	+		+		
Metapseudomonas resinovorans	Pseudomonadales	WP_016492312			+		
Shewanella algae	Alteromonadales	WP_156018594			+		
Thalassomonas viridans	Alteromonadales	WP_044841159			+		
**Bacillota**							
Oceanobacillus piezotolerans YLB-02	*Bacillales*	WP_121523662	+		+		
Mesobacillus campisalis	Bacillales	WP_052948620	+		+		
Paucilactobacillus vaccinostercus	Lactobacillales	WP_083483783	+		+		
Selenobaculum gbiensis	Selenomonadales	WP_147669261			+		
Megasphaera cerevisiae	Veillonellales	WP_048513802			+		
**Planctomycetota**							
Bremerella cremea	Pirellulales	WP_105358529			+		
Novipirellula maiorica	Pirellulales	WP_008692238			+		
Neorhodopirellula lusitana	Pirellulales	WP_283435124			+		
Rubinisphaera brasiliensis	Planctomycetales	WP_013630173		+	+		
Rubinisphaera italica	Planctomycetales	WP_146503709			+		
**Fibrobacterota**							
Hallerella succinigenes	Fibrobacterales	WP_100425063			+		
Fibrobacter sp. UWH4	Fibrobacterales	WP_072980470			+		

Table S2 - Representative PbfG sequences and composition of the corresponding degradative gene clusters. The PbfG proteins belong to the pf00107 family, A cladogram representing the relationships between these sequences is provided in Figure S2A.

Organism	Order	Accession ID	Other genes in cluster				
			phnW	*pbfD*	*phnX*	*phnY*	*phnA*
**Actinomycetota**							
Cellulosimicrobium protaetiae	Micrococcales	WP_154797266		+	+		
Microbacterium foliorum 122	Micrococcales	WP_079113449		+	+		
Microbacterium esteraromaticum	Micrococcales	WP_259363394		+	+		
Microterricola viridarii	Micrococcales	WP_082717095		+	+		
Frigoribacterium endophyticum	Micrococcales	WP_341777359		+	+		
Brevibacterium aurantiacum	Micrococcales	WP_208649196		+	+		
Nocardia farcinica	Mycobacteriales	BAD58375		+	+		
Rhodococcus ruber	Mycobacteriales	WP_203011493		+	+		
Mycobacterium paraense	Mycobacteriales	WP_085102210			+		
Mycolicibacterium litorale	Mycobacteriales	AQT82760			+		
Pseudonocardia broussonetiae	Pseudonocardiales	WP_172164990		+	+		
Spiractinospora alimapuensis	Streptosporangiales	WP_236542728				+	+
Aeromicrobium chenweiae	Propionibacteriales	WP_108579078		+	+		
**Bacteroidota**							
Filimonas lacunae	Chitinophagales	WP_076379520			+		
Mucilaginibacter kameinonensis	Sphingobacteriales	WP_121808920			+		
Chondrinema litorale	Cytophagales	WP_284687237			+		
Echinicola rosea	Cytophagales	WP_137402342		+	+		
**Planctomycetota**							
Aureliella helgolandensis	Pirellulares	WP_197355854		+	+		
Botrimarina mediterranea	Pirellulares	QDV73922			+		
Rubripirellula lacrimiformis	Pirellulares	WP_145176407		+	+		
Gimesia maris	Planctomycetales	WP_002648694	+	+	+		
Gimesia chilikensis	Planctomycetales	WP_145438294	+	+	+		
Caulifigura coniformis	Planctomycetales	WP_145034394				+	+
Thalassoglobus polymorphus	Planctomycetales	WP_231739950		+	+		
Maioricimonas rarisocia	Planctomycetales	WP_197444149		+	+		
Urbifossiella limnaea	Gemmatales	WP_145243951		+	+		
Singulisphaera acidiphila	Isosphaerales	WP_015244017		+	+		
**Verrucomicrobiota**							
Rubritalea squalenifaciens	Verrucomicrobiales	SHK12509				+	+
Brevifollis gellanilyticus	Verrucomicrobiales	WP_146852304				+	+
Verrucomicrobium sp. BvORR106	Verrucomicrobiales	WP_051945821				+	+
**Gemmatimonadota**							
Gemmatimonadetes bacterium RS822	Gemmatimonadales	MBN63099		+		+	+
Gemmatimonadaceae bacterium NP81	Gemmatimonadales	MBQ39625				+	+
Gemmatimonadaceae bacterium SP3091	Gemmatimonadales	MBI91831				+	+

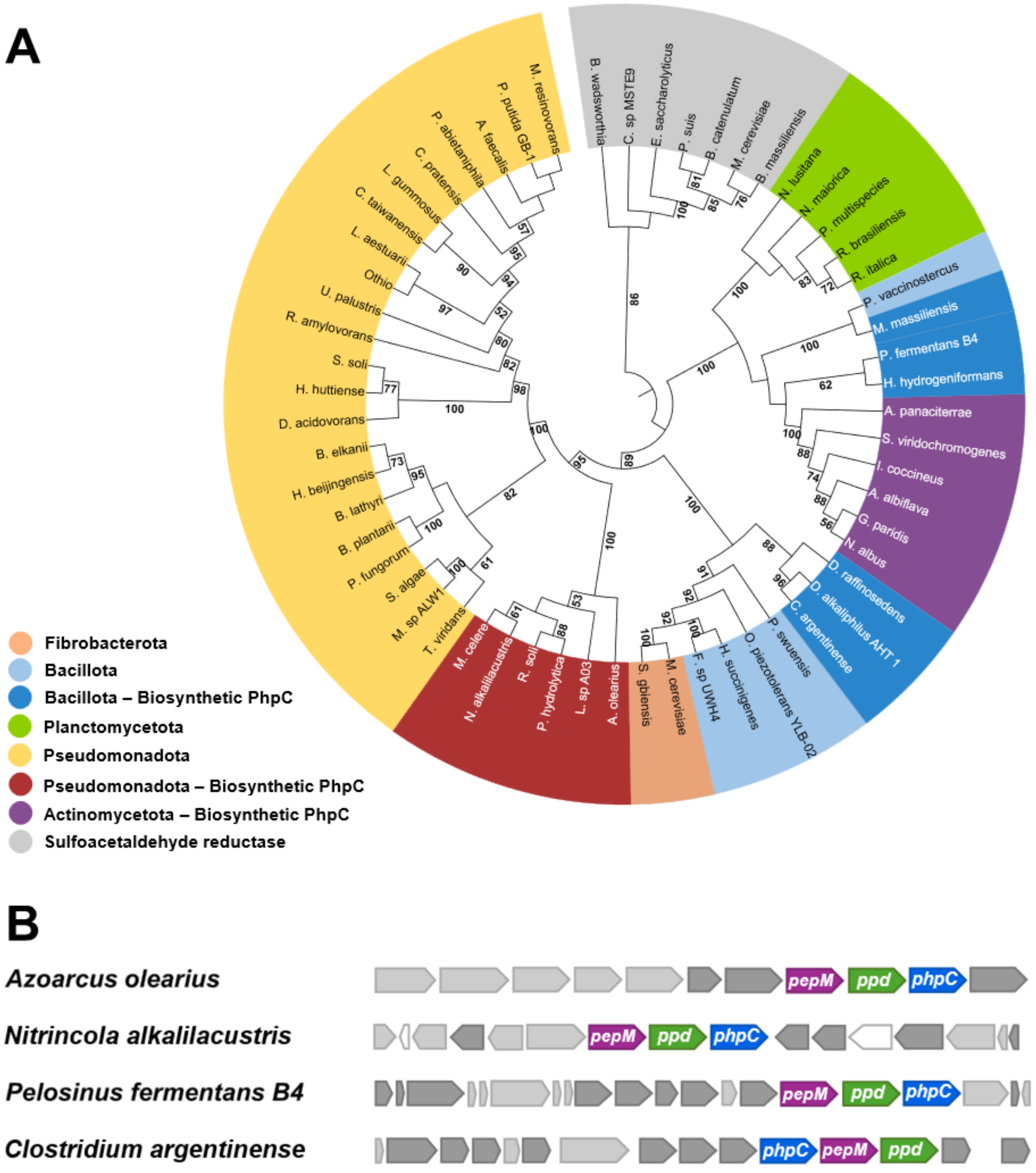

Figure S1: (A) Circular cladogram showing the phylogenetic distribution and the relationships of the degradative PhpC sequences listed in Table S1 and selected homologous enzymes. In particular, the cladogram includes some biosynthetic PhpC sequences from Actinomycetes (Shao et al., 2008; Zhang et al., 2022) and some other PhpC homologs encoded in putative biosynthetic clusters from Pseudomonadota and Bacillota (see below), as well as the structurally related sulfoacetaldehyde reductases, which were used as the outgroup. The tree *does not* include some close PhpC homologs whose catalytic activities remain uncharacterized and whose genes do not belong to clusters for phosphonate metabolism; the most notable example is PsrA from *Burkholderia cenocepacia* (~70% identical to PhpC from *P. fungorum*), a hypothetical reductase which has been implicated in the

detoxication of membrane lipid peroxidation products (Naguib et al., 2022). The tree was built based on a multiple sequence alignment, as described in the Methods. The tree branching mostly corresponds to the different phyla, with limited suggestions for inter-phyla horizontal transfer of the *phpC* gene. Branches corresponding to putative biosynthetic and degradative PhpC sequences are interspersed throughout the tree, suggesting that the physiological role of the enzyme is dictated primarily, if not exclusively, by the metabolic context rather than by obvious structural differences. Of note, however, the biosynthetic PhpC enzymes from *Streptomyces* species are reportedly monomeric (Shao et al., 2008), whereas the degradative PhpC from *P. fungorum* is dimeric (see Figure S3 below). (B) Some apparently biosynthetic clusters from Pseudomonadota (*A. olearius* and *N. alkalilacustris*) and Bacillota (*P. fermentans* and *C. argentinense*) that include *phpC*-type genes (in blue) together with phosphonopyruvate decarboxylase (*ppd*, in green; the gene is sometimes also called *aepY*) and phosphoenolpyruvate phosphomutase (*pepM*, in pink). The products of the *phpC* genes shown in panel B correspond to some of the sequences highlighted in dark red and dark blue in panel A. In addition to Actinomycetota, Pseudomonadota and Bacillota, biosynthetic clusters containing *phpC* associated with *pepM* and *ppd* are found in numerous bacterial phyla, including Bacteroidota, Cyanobacterota, Synergestota, Thermodesulfobacterota, Balneolota and Elusimicrobiota.

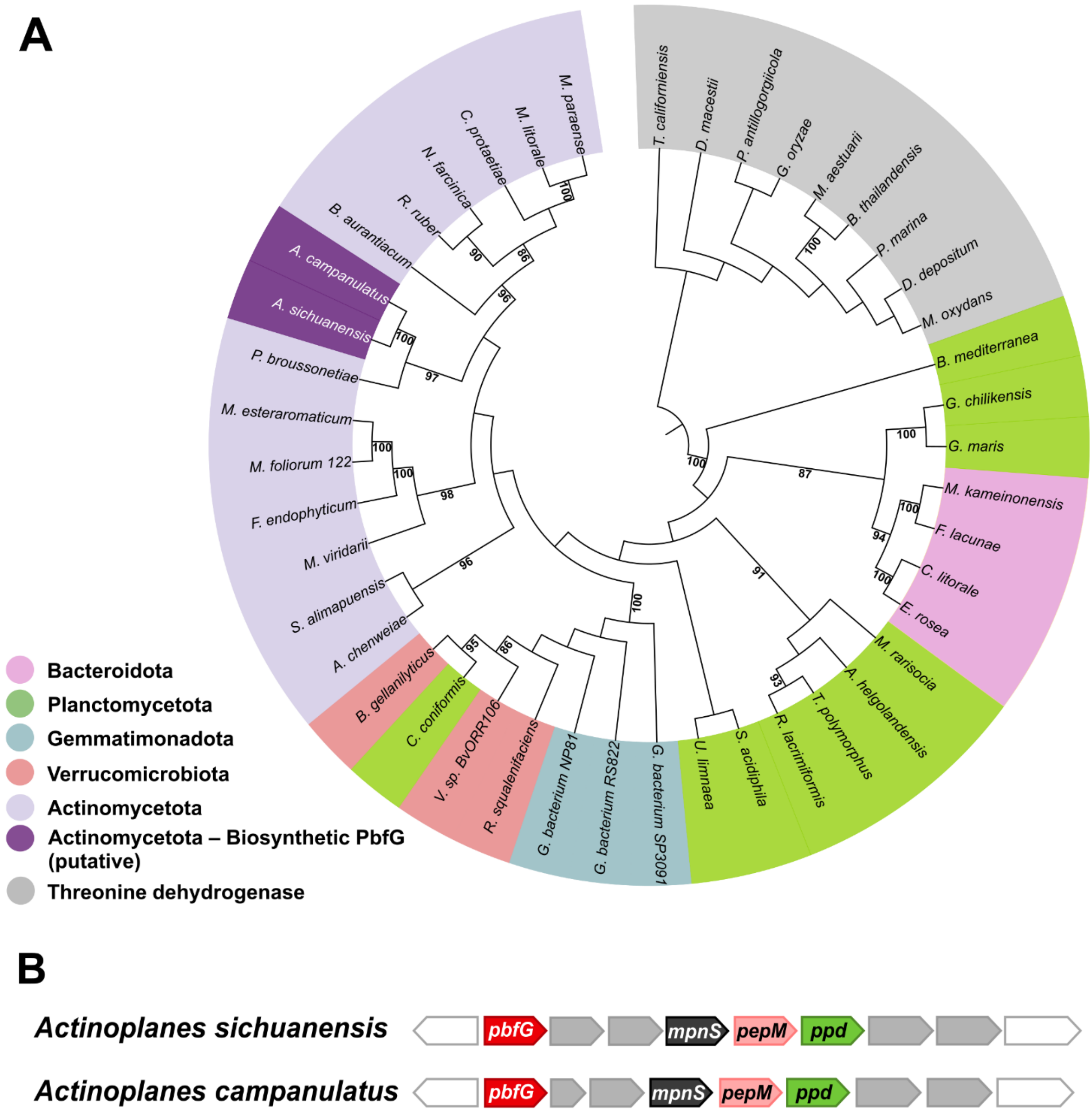

Figure S2: (A) Circular cladogram showing the phylogenetic distribution and the relationships between the PbfG sequences listed in Table S2 and selected homologous enzymes. Threonine dehydrogenases were used as the outgroup, confirming their clear divergence from the PbfG sequences. The relatively low bootstrap values and the heterogeneity observed among Bacteroidota and Planctomycetota sequences suggest either a more recent divergence of these sequences or possible events of horizontal gene transfer. (B) Two putative biosynthetic gene clusters with *pbfG*-like genes, from *Actinoplanes* species (phylum Actinomycetota). These putative biosynthetic *pbfG* genes correspond to the branch highlighted in purple in panel A and may participate in the biosynthesis of methylphosphonate, as suggested by the presence of *mpnS* homologs in the clusters (Born et al., 2017; Metcalf et al., 2012).

As shown in panel A, the PbfG sequences from *Actinoplanes* fall into the broader branch of the Actinomycetota sequences, consistent with a relatively recent emergence of biosynthetic PbfG enzymes from a degradative ancestor.

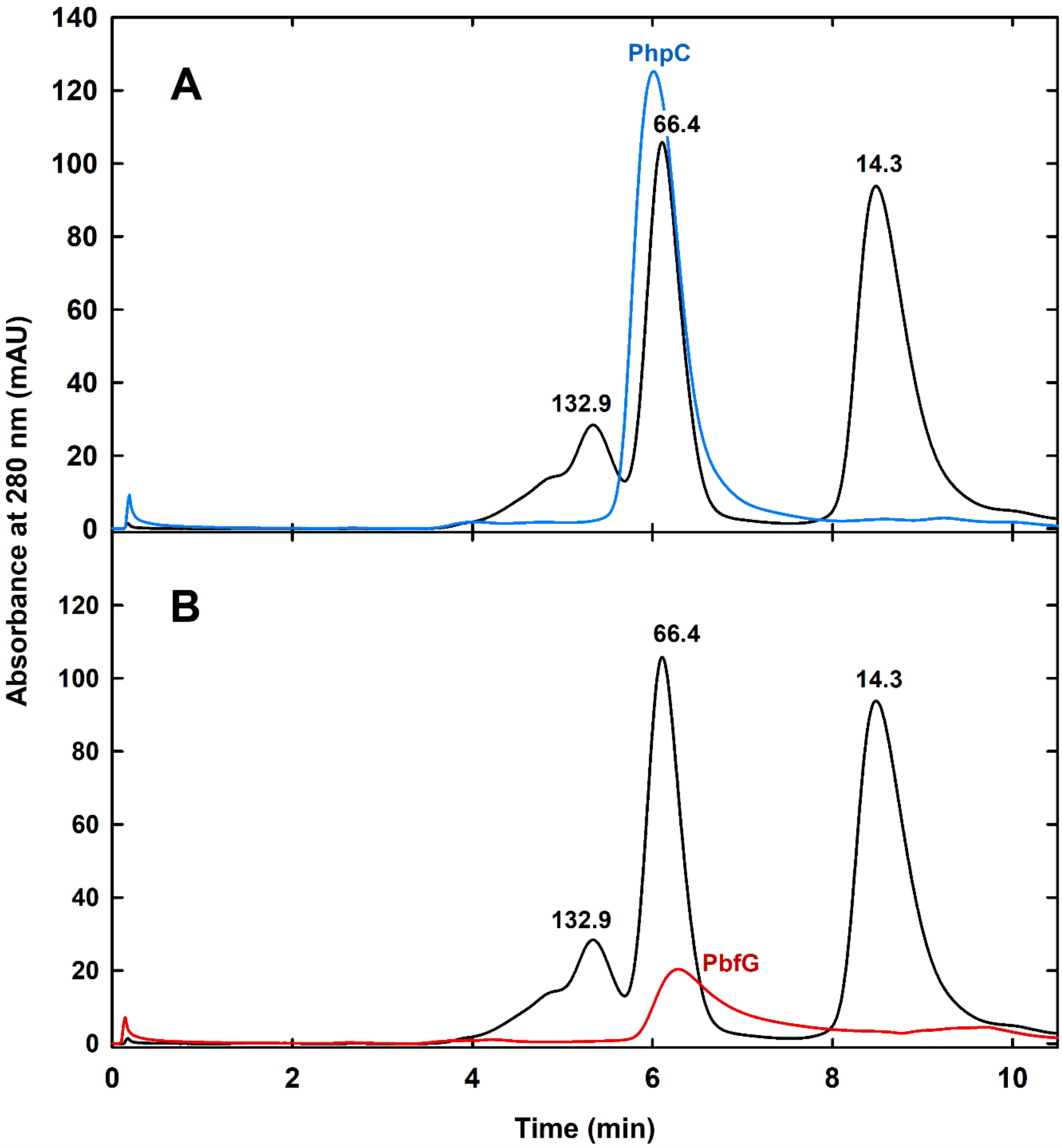

Figure S3: Estimating the oligomerization state of recombinant PhpC from *P. fungorum* and PbfG from *C. protaetiae* by size-exclusion chromatography. (A) The chromatogram of PhpC (blue), compared with that of three molecular mass standards (black), indicates an apparent molecular mass of 78 kDa (versus a calculated monomeric mass of 40.8 kDa). (B) Likewise, the chromatogram for PbfG (red), suggests an apparent molecular mass of 64.2 kDa (versus a calculated monomeric mass of 36.9 kDa). Size-exclusion chromatography experiments were carried out as described in the Methods section of the main text.

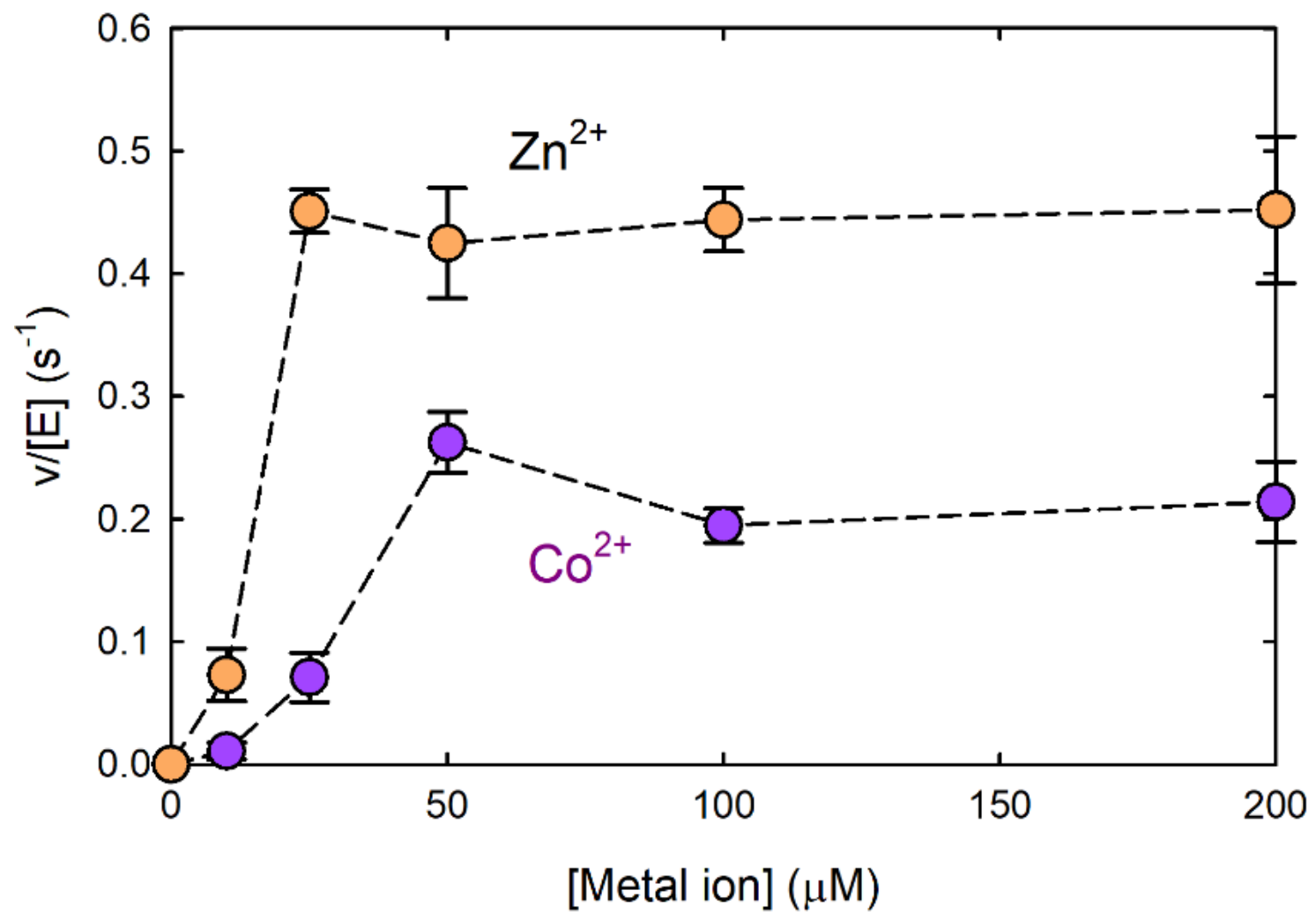

Figure S4: Reactivation of EDTA-treated PhpC by increasing concentrations of either zinc or cobalt ions. As detailed in the Methods, a PhpC stock was inactivated by prolonged incubation with excess EDTA, followed by a desalting step. Reactivation was then assessed by adding the EDTA-treated enzyme to a reaction mixture that contained 5 mM HEP, 1.5 mM NAD^+ and various concentrations of either $ZnCl_2$ (orange circles) or $CoCl_2$ (purple circles). Other conditions: 50 mM TEA plus 20 mM Bis-Tris, pH 8.0, 25°C.

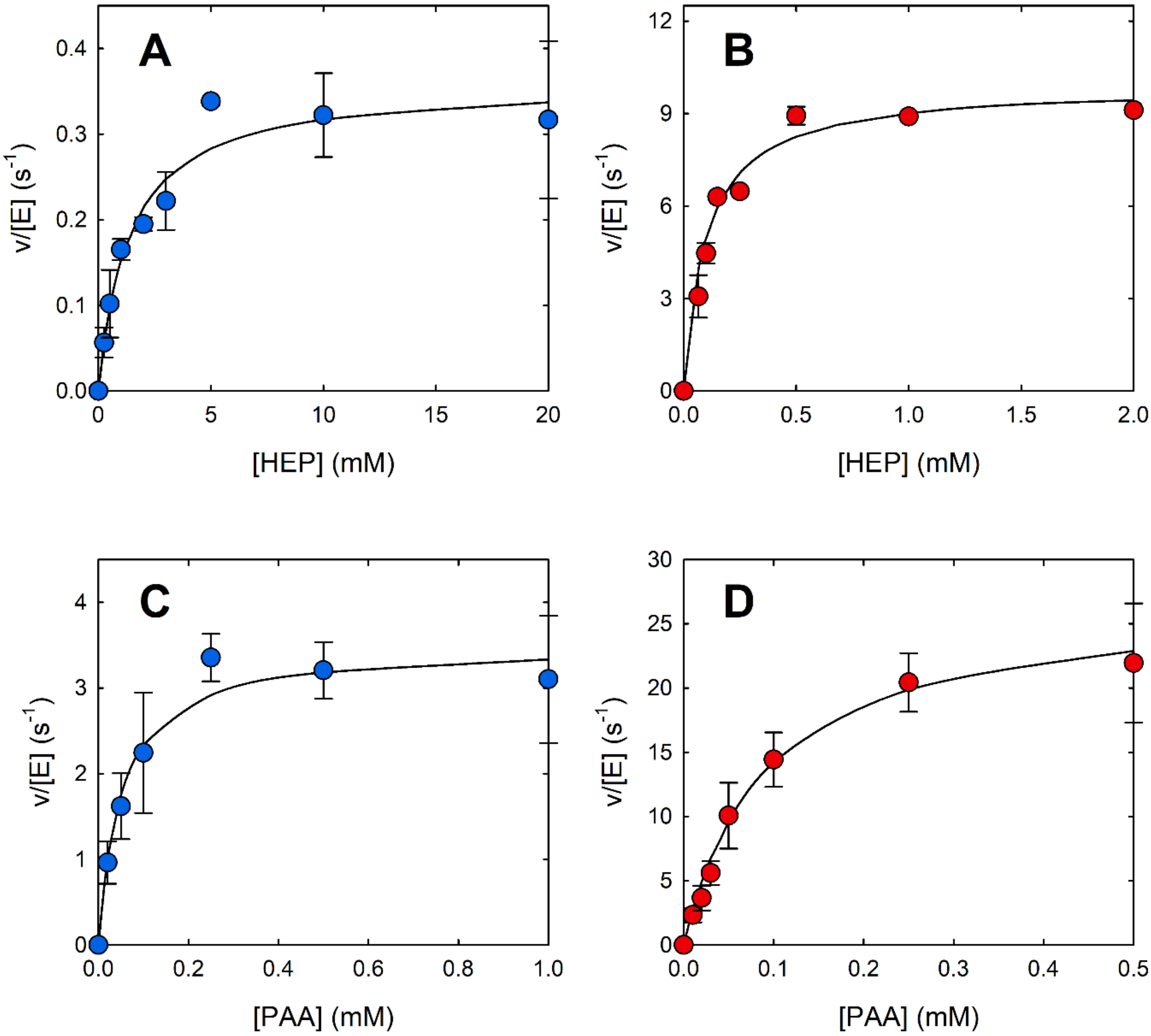

Figure S5: Dependence of the activities of PhpC (blue) and PbfG (red) on the concentration of their substrates – AEP for the forward reaction and PAA for the reverse reaction. Assays were performed in 50 mM TEA-Cl buffer, pH 8.0, 25°C. For the HEP dehydrogenase reaction (panels A and B), the NAD^+ concentration was fixed at 1.5 mM. For the PAA reductase reaction (panels C and D), the NADH concentration was fixed at 0.25 mM. Data points represent the mean (±SD) of three technical replicates, and the solid curves correspond to nonlinear least-squares fittings of the data to the Michaelis-Menten equation (Eq. 1 in the main text). The resulting kinetic parameters are reported in Table 1 of the main text.

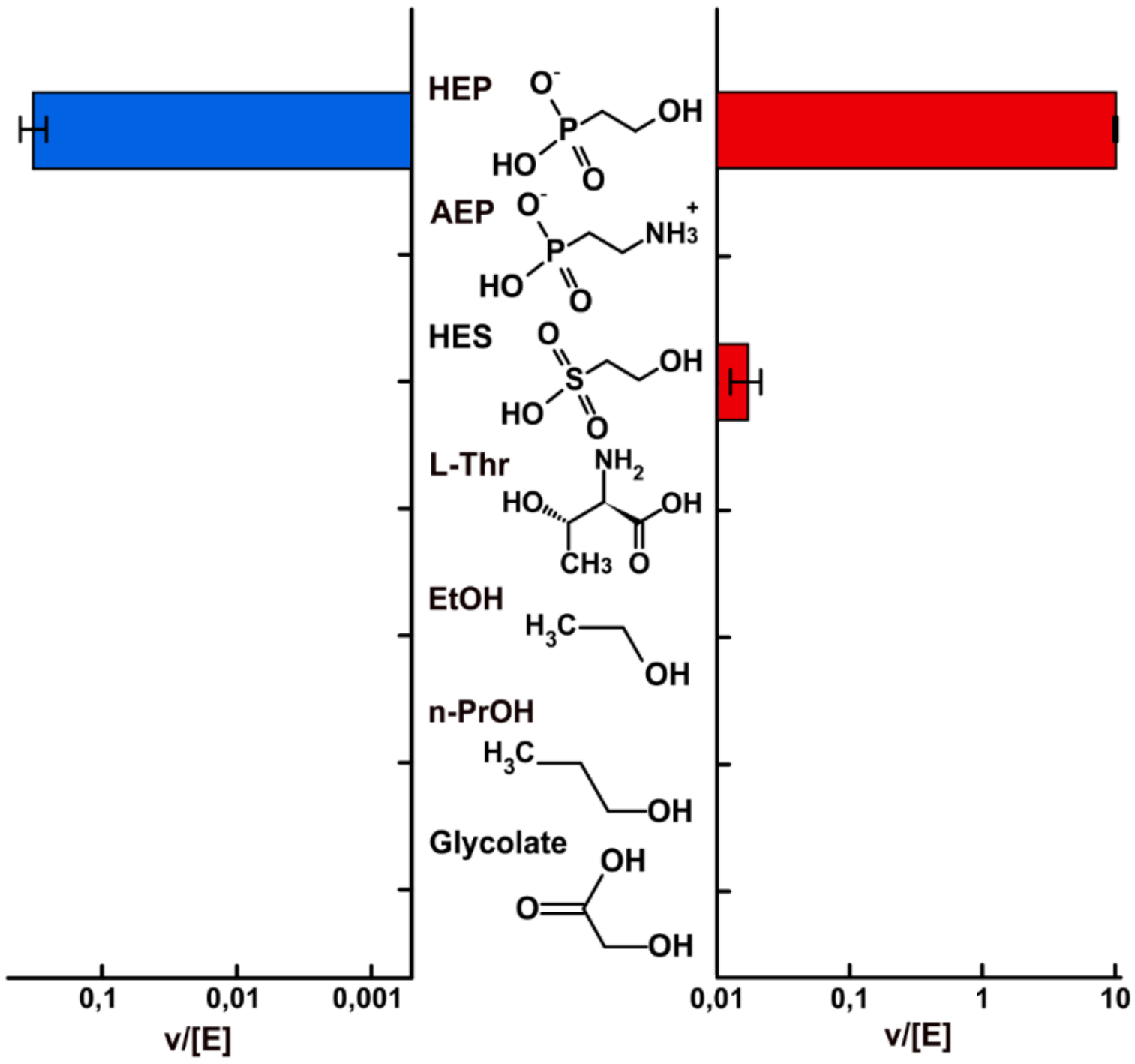

Figure S6: Activity of PhpC and PbfG toward selected HEP analogs. The bar graph shows the activity of PhpC (0.33 µM; blue) and PbfG (0.1 µM; red) towards HEP and several potential substrates (5 mM in each case). HES denote isethionate (2-hydroxyethanesulfonate). Note that the horizontal axis is logarithmic. Although isethionate was clearly oxidized by PbfG, the rate of this reaction was 250-fold lower than that observed with HEP under the same conditions.

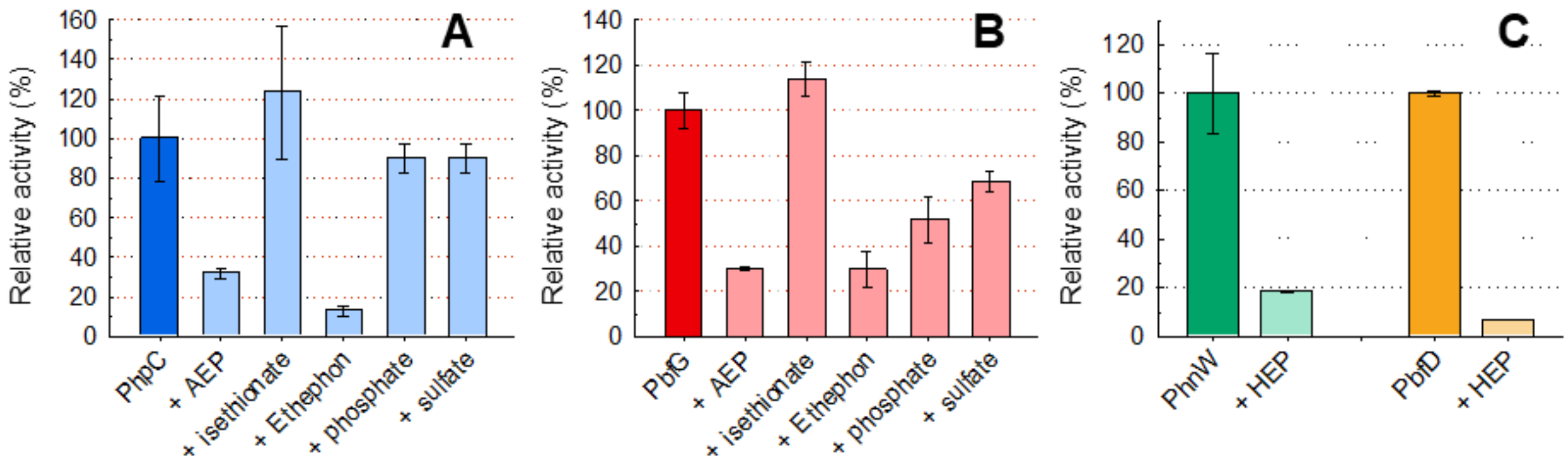

Figure S7: Results of inhibition assays. (A) Effect of AEP (10 mM), isethionate, Ethephon (2-chloroethylphosphonate), phosphate and sulfate (15 mM) on the activity of PhpC (0.33 μM) in the presence of 0.5 mM HEP. Bars represent the percent activity (mean ± SD, calculated on three technical replicates) relative to the absence of inhibitor (darker bar on the left). (B) Effect of the same anionic compounds tested in panel A on the activity of PbfG (0.1 μM). (C) Inhibitory effect of HEP (10 mM) on the activities of PhnW from *Vibrio splendidus* (Zangelmi et al., 2021) and PbfD from *Mariniblastus fucicola* (Zangelmi et al., 2023). In these assays, AEP was used at concentrations close to the K_M of each enzyme (i.e., the AEP concentration was 0.5 mM for reactions involving PhnW and 2 mM for reactions involving PbfD).

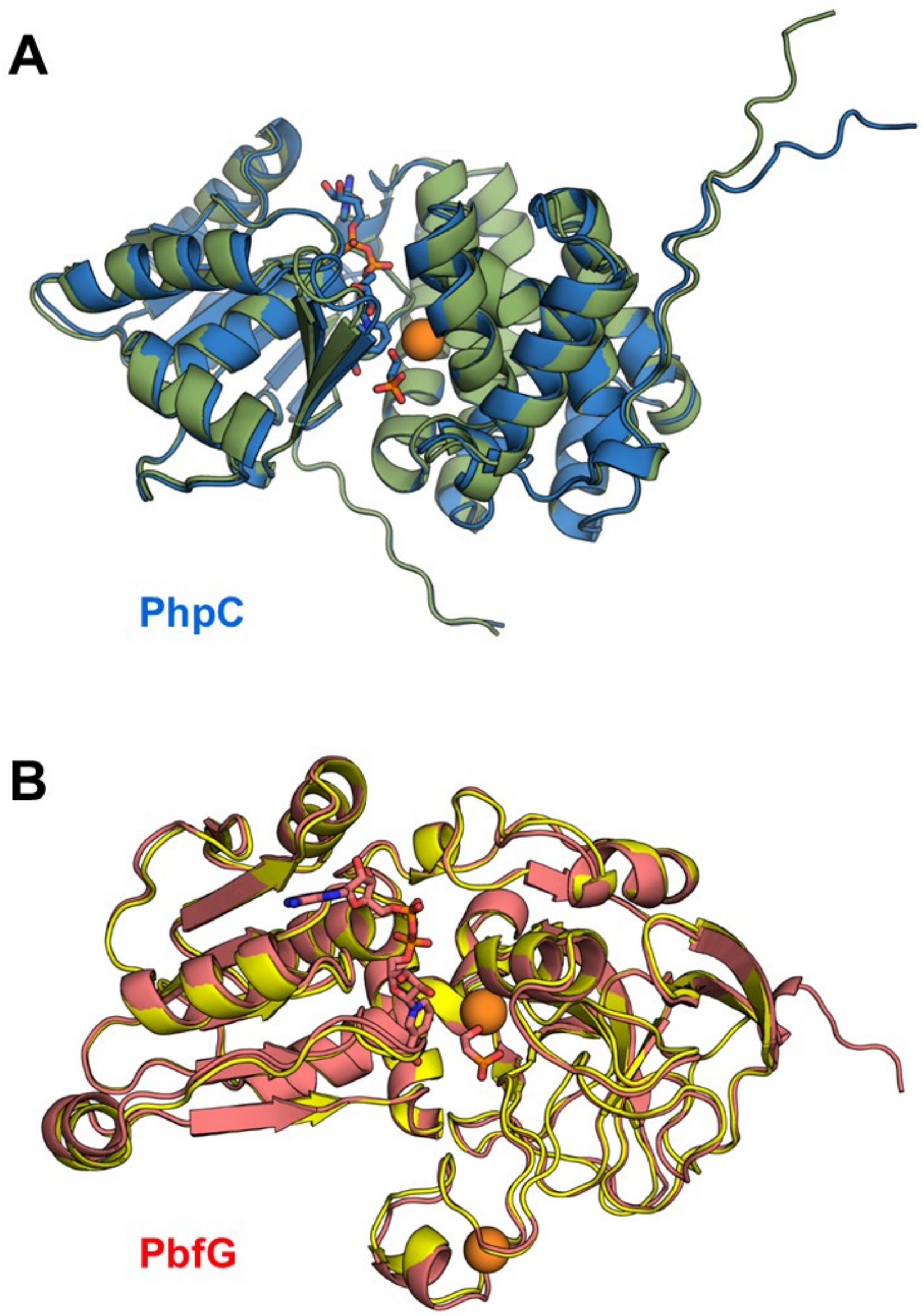

Figure S8: Minimal effect of ligands on the three-dimensional structures of monomeric PhpC and PbfG predicted by Alphafold3. (A) Structural alignment of the ligand-free *P. fungorum* PhpC model (green) with the fully liganded PhpC model containing NAD, Zn^{2+} and HEP (blue). Both models showed high global confidence, reaching ipTM/pTM values of 0.96/0.95 for the liganded PhpC. Comparison of the two models revealed only minimal backbone differences, with a Cα RMSD of 0.529 Å. The fully liganded monomer model aligned also very well with the structure of PhpC in the fully liganded dimeric model (Cα RMSD = 0.173 Å). (B) Structural alignment of the ligand-free *C. protaetiae* PbfG model (pink) with the fully liganded PbfG model (containing NAD + Zn^{2+} + Zn^{2+} + HEP) (yellow). Also in this case, both models showed high global confidence, with the liganded model in particular reaching ipTM/pTM values of 0.97/0.95. The structural alignment yielded a Cα RMSD of 0.508 Å. The fully liganded model shown here aligned also well with the structure of PbfG in the fully liganded dimeric model (Cα RMSD = 0.900 Å). In all cases the structural alignments were generated using PyMOL (DeLano, 2002).

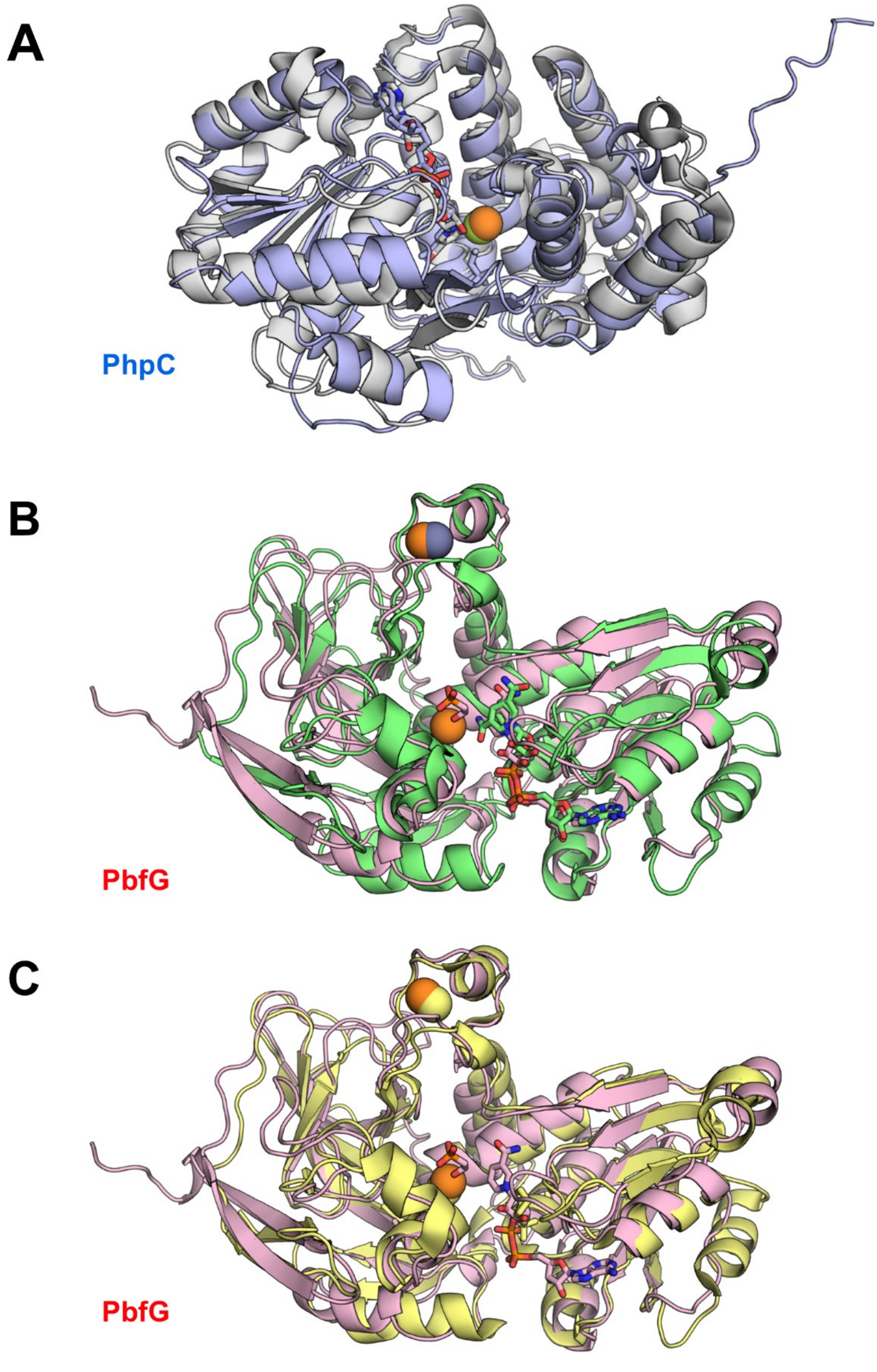

Figure S9: Comparison of the AlphaFold3 models of PhpC and PbfG (fully liganded) with the experimental structures of their closest homologs, as identified by Foldseek (van Kempen et al., 2024). (A) Structural alignment of the *P. fungorum* PhpC model (blue) with the X-Ray structure of sulfoacetaldehyde reductase from *B. kashiwanohense* (PDB 6JKP; gray) (Zhou et al., 2019) (Cα RMSD = 1.346 Å). (B). Structural alignment of the *C. protaetiae* PbfG model (pink) with threonine

dehydrogenase from *Pyrococcus horikoshii* (PDB 2DFV; green) (Ishikawa et al., 2007) (Cα RMSD of 1.540 Å). (C) Structural alignment of PbfG model (pink) with the X-Ray structure of threonine dehydrogenase from *Thermus thermophilus* (PDB 2DQ4; yellow) (Cα RMSD = 1.663 Å). In addition to the relatively low Cα RMSD values, both PhpC and PbfG AF3 models placed the bound NAD and Zn^{2+} ions in positions closely matching those observed in their respective structural homologs, supporting the reliability of the active site architectures.

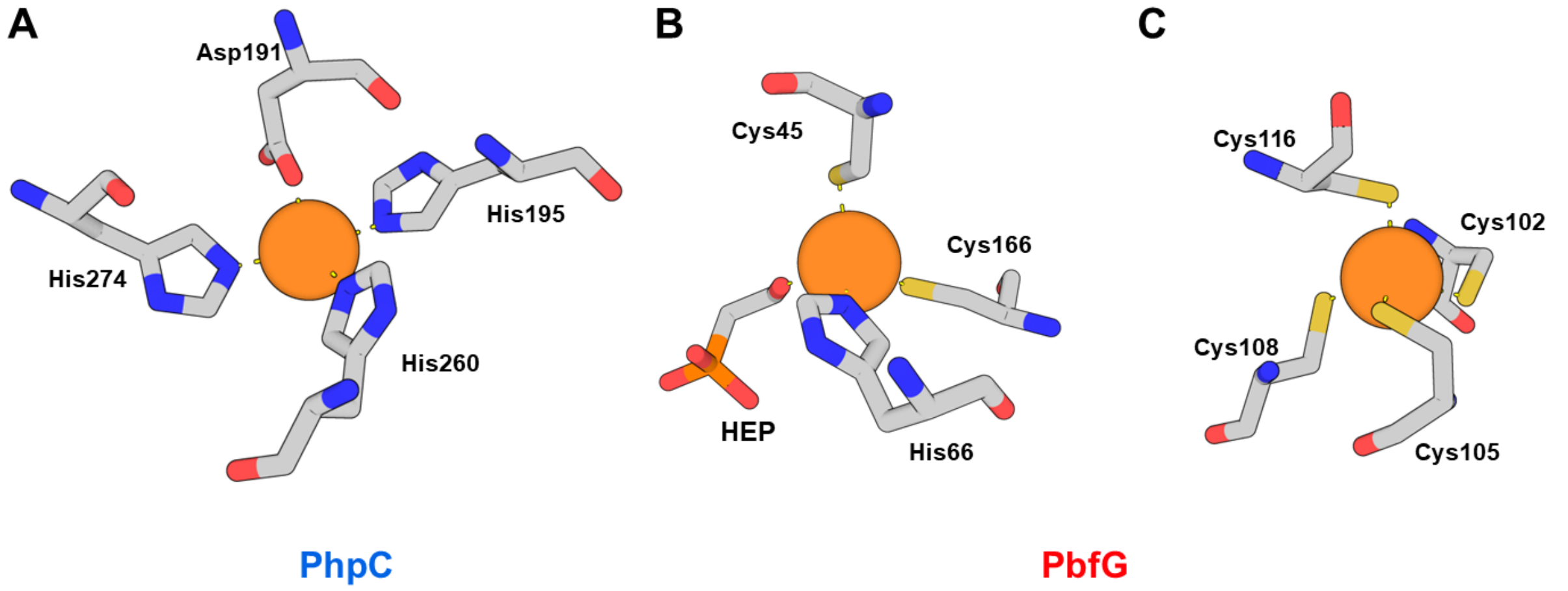

Figure S10: Predicted metal binding sites in PhpC from *P. fungorum* and PbfG from *C. protaetiae*. (A) Predicted catalytic Zn^{2+} (orange) bound near the active site of *P. fungorum* PhpC. The Zn^{2+} coordinating amino acids are conserved in most of the sequences in Table S1; in the few exceptions (mostly from Planctomycetota) His195 is replaced by Gln. (B) Predicted catalytic Zn^{2+} bound in the active site of *C. protaetiae* PbfG. In the AF3 model, the metal ion is coordinated by Cys45, His66, Cys116 and the hydroxyl oxygen of HEP. The three protein residues are conserved in all the sequences listed in Table S2. (C) Predicted structural Zn^{2+} bound in the PbfG AF3 model. The model placed the metal ion away from the active site and coordinated by four cysteine residues in a canonical tetrahedral geometry. An analogous structural Zn^{2+} binding site is found in threonine dehydrogenase (Ishikawa et al., 2007) (see Figure S9) and in other pf00107 dehydrogenases (Bock et al., 2016; Thomas et al., 2013). The four coordinating cysteines are conserved in all sequences in Table S2, except those from Bacteroidota, where the structural metal binding site appears to be absent.

.

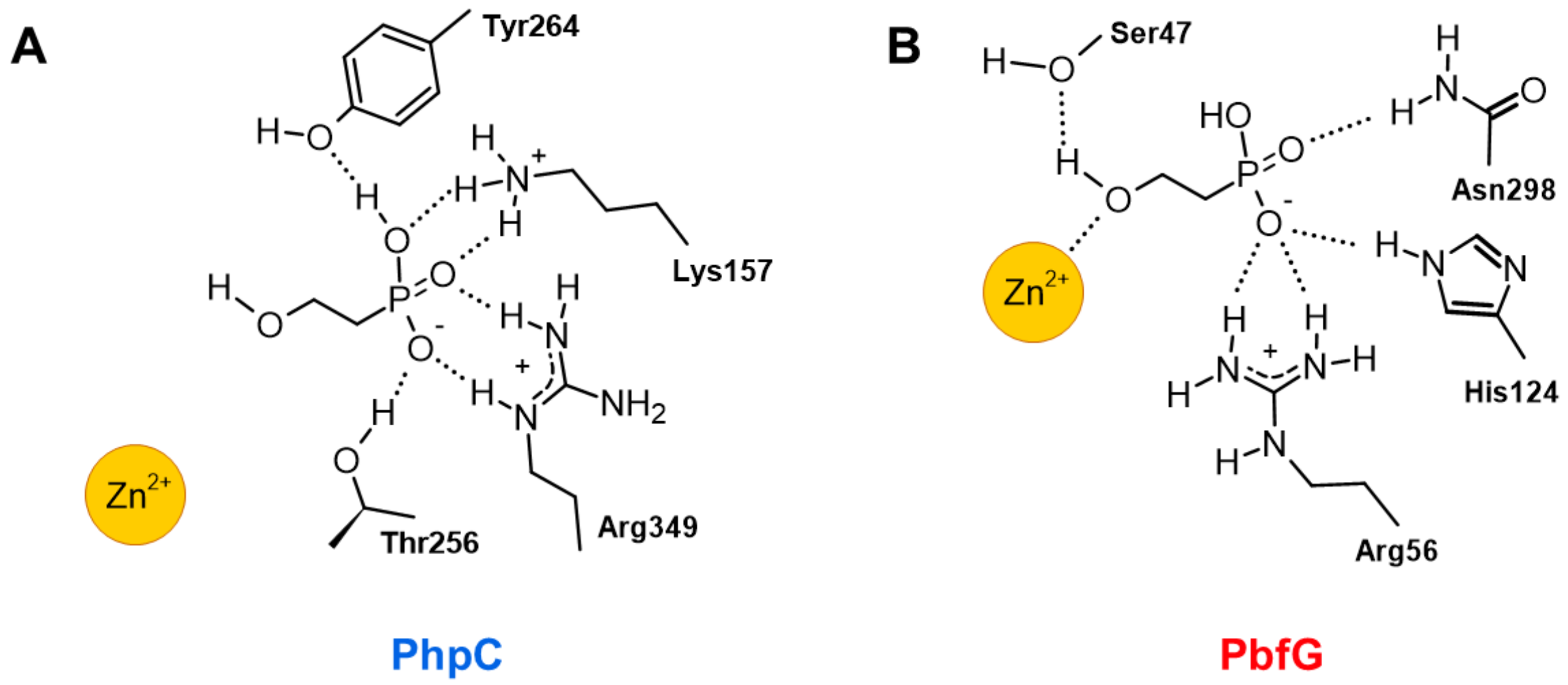

Figure S11: Schematic, two-dimensional view of the predicted interactions of HEP in the active sites of PhpC and PbfG. (A) Predicted interactions of HEP in the PhpC active site. In the AF3 model, no protein side chain capable of acting as a general base is located near the hydroxyl group of HEP. Furthermore, the distance between the hydroxyl oxygen of HEP and the Zn^{2+} ion is approximately 3.5 Å, too long to support direct coordination. (B) Interactions of HEP in the active site of the PbfG AF3 model. In this case, the predicted distance between the hydroxyl oxygen and the metal ion is approximately 2.3 Å, consistent with direct, albeit relatively weak, coordination. In addition to the hydrogen bonds shown here, the phosphonate group of HEP may also form a long-range electrostatic interaction with the guanidinium group of Arg288 from the other subunit (see Figure 6B in the main text).

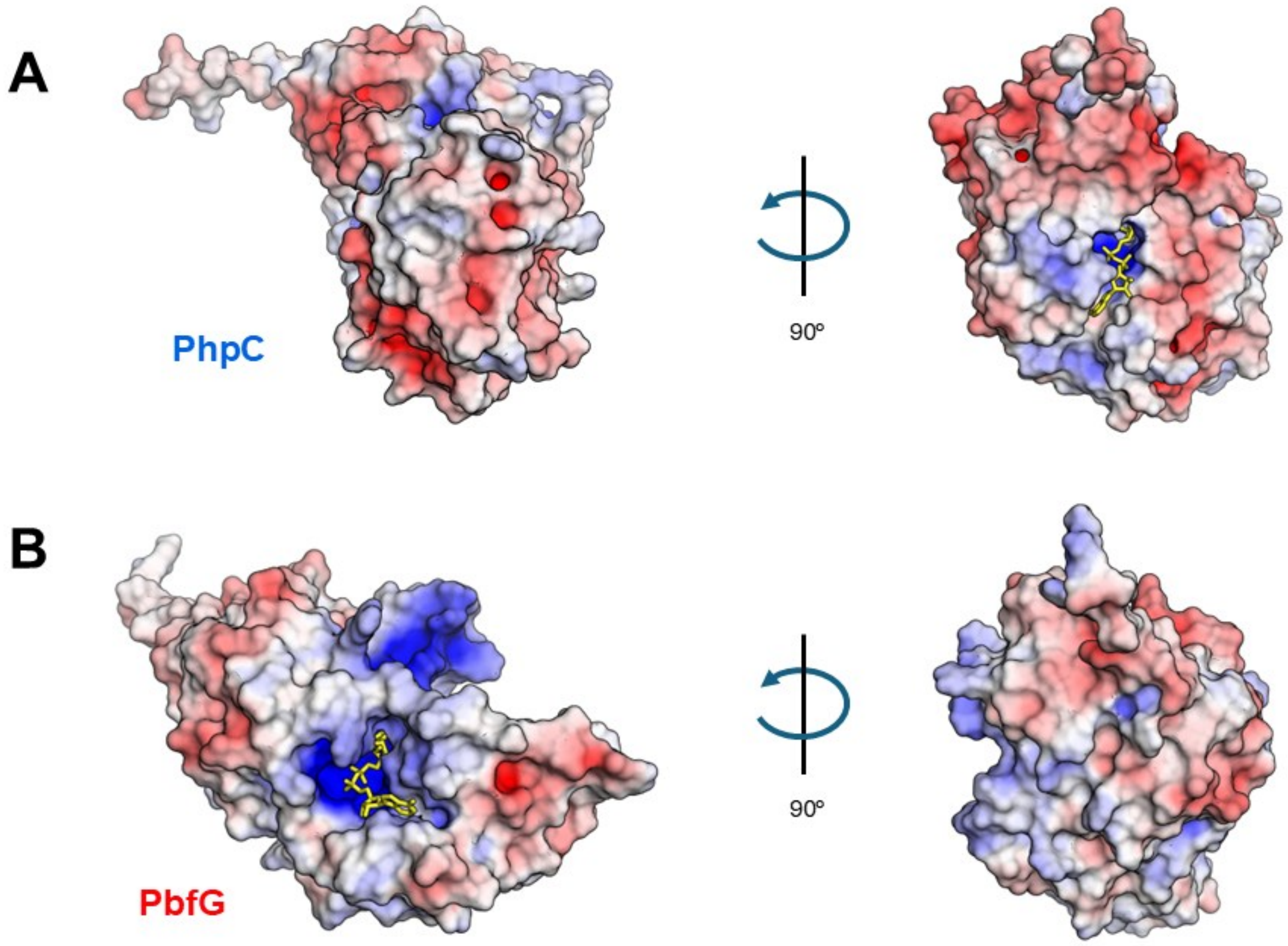

Figure S12: Electrostatic surfaces of PhpC from *P. fungorum* and PbfG from *C. protaetiae.* (A) On the left, the PhpC monomer is shown oriented as in Figure 6A of the main text; on the right, the same monomer is shown rotated by 90° around the vertical axis, to better visualize the active site entrance. (B) Electrostatic surface of the liganded PbfG monomer, shown in the same two orientations as in panel A. A positively charged surface cleft extends towards the catalytic Zn^{2+} and may facilitate binding of the negatively charged phosphonate group of HEP. The electrostatic surfaces of the monomers were calculated using PyMOL (DeLano, 2002) after removing the ligands, which were subsequently reintroduced for display.

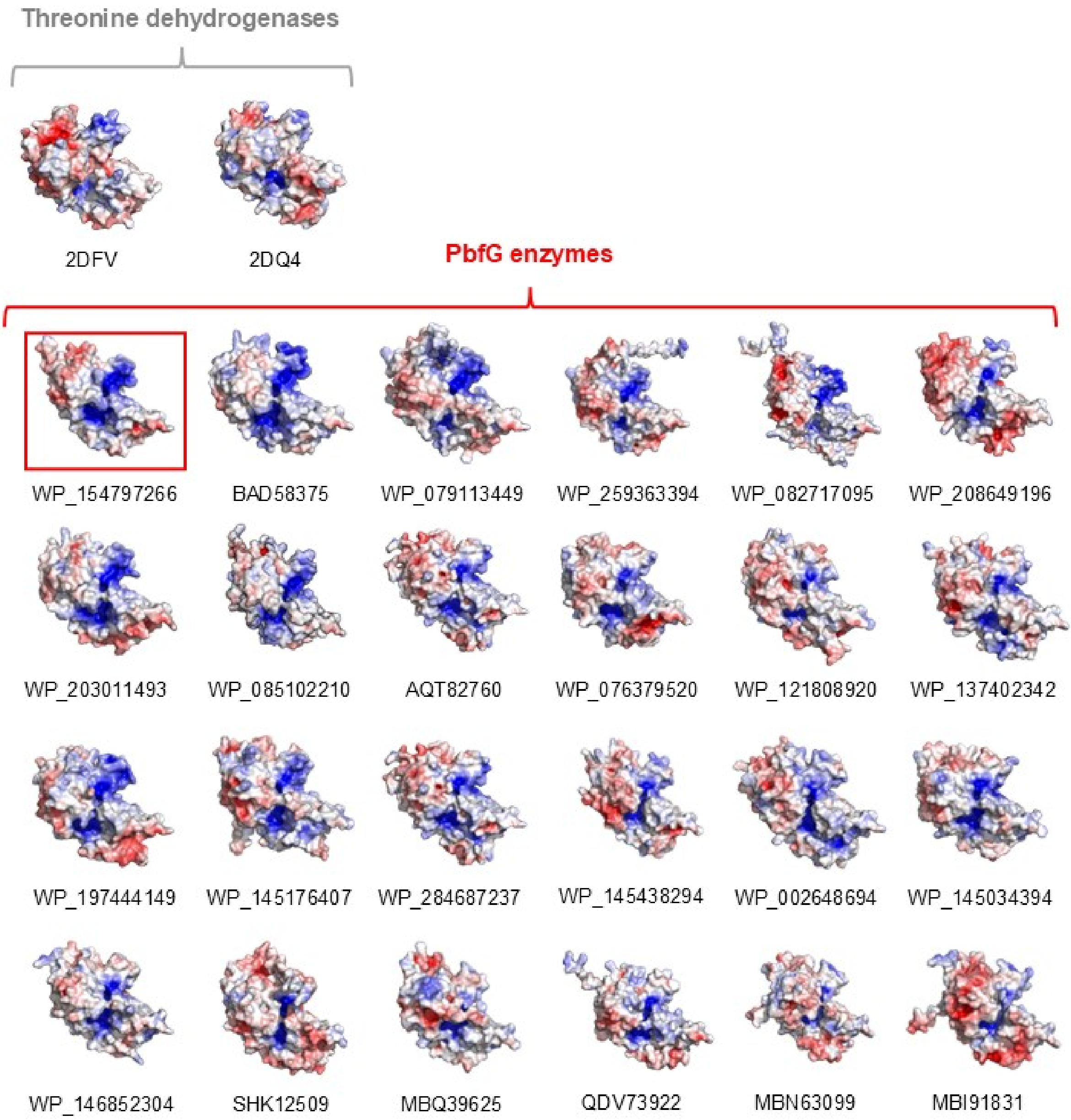

Figure S13: Conservation of the positively charged surface cleft of PbfG. The electrostatic surface of the model PbfG from *C. protaetiae* (WP_154797266; structure boxed in red) is compared with those of two threonine dehydrogenases (from *Thermus thermophilus* - 2DQ4 - and *Pyrococcus horikoshii* - 2DFV) and with those of 23 other PbfG-type proteins listed in Table S2. The corresponding NCBI accession number is shown below each model.

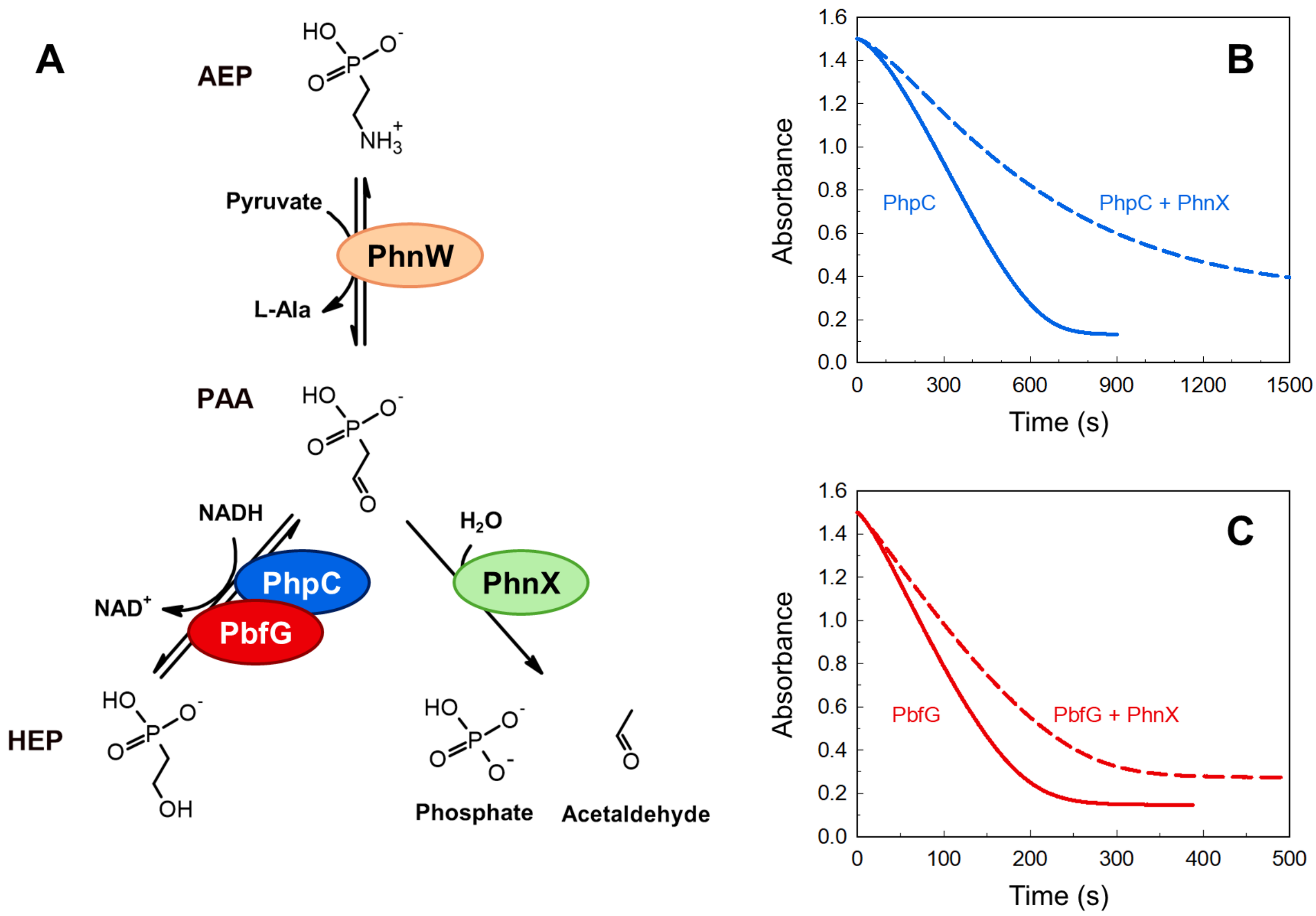

Figure S14: Competition between the HEP dehydrogenases and PhnX for the processing of PAA. (A) Schematic representation of the experimental setup, in which PAA (generated by transamination of AEP) can be either converted to HEP by PhpC or PbfG or hydrolyzed to acetaldehyde and phosphate by PhnX. As the PAA reduction can be monitored spectrophotometrically at 340 nm, it is also possible to assess how much the observed kinetics change when PhnX is present in the reaction mixture. Experiments were conducted in 50 mM TEA buffer, pH 8.0, in the presence of 250 μM NADH, 2 mM AEP, 2 mM pyruvate and 1 mM $MgCl_2$. (B) Rate of NADH consumption by PhpC (0.5 μM) with or without the addition of equimolar amounts of PhnX from *Vibrio splendidus* (Zangelmi et al., 2021). In the presence of PhnX, the observed rate of NADH consumption decreased by two to threefold, indicating that PhpC and PhnX were competing for PAA with comparable efficiencies. Indeed, as a first approximation and under steady-state conditions, PAA should partition between the dehydrogenase and the hydrolase reactions according to the respective k_{cat}/K_M values (for PhpC, k_{cat}/K_M = 68000 $M^{-1}s^{-1}$, see Table 1 in the main text; for PhnX, k_{cat}/K_M = 138000 $M^{-1}s^{-1}$, data not shown). (C) Rate of NADH consumption by PbfG (0.5 μM) with or without the addition of equimolar amounts of PhnX. Consistent with its higher k_{cat}/K_M (290000 $M^{-1}s^{-1}$; see Table 1 in the main text), PbfG competed more efficiently with PhnX, and in the presence of PhnX the rate of NADH consumption decreased by only 30%.

Supplementary scheme

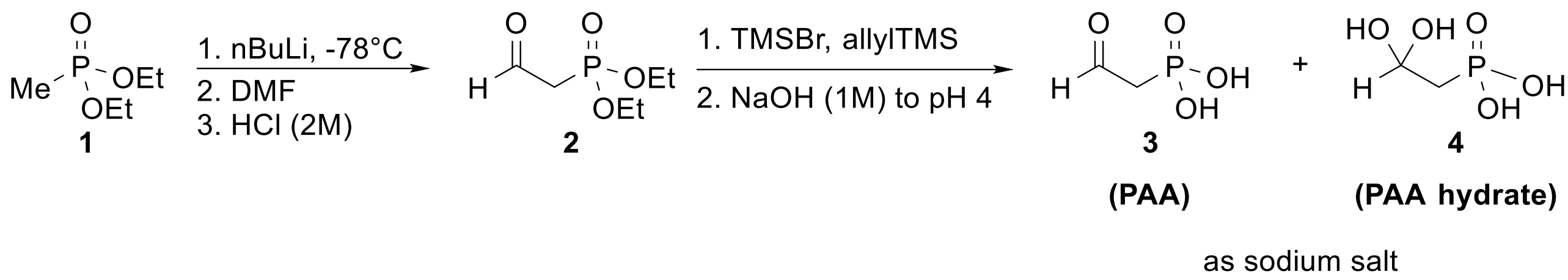

Scheme S1: chemical synthesis of PAA.

Supplementary Data

Data S1 - Recorded ^{1}H, ^{13}C and ^{31}P NMR spectra of all the compounds synthesized for the preparation of PAA. All assignments, solvents and device parameters are given in the Materials and Methods section, and partly on top of each picture. The first spectrum shown in each series is the full ^{1}H NMR spectrum. Expansions are depicted where they were regarded as necessary. Then the ^{31}P NMR spectrum and the ^{13}C NMR spectrum are depicted in the same manner. The x-axis is given in ppm and the peak labels are in Hertz (Hz) for all shown spectra. Structures are always given on top of the full ^{1}H NMR spectrum. Integrals are denoted below the x-axis where they were regarded as necessary and the integration range is marked. The numbering and order of compounds is in accordance with the numbering in Scheme S1.

^{1}H NMR (400.27 MHz, $CDCl_3$) **of diethyl 2-oxoethylphosphonate (2):**

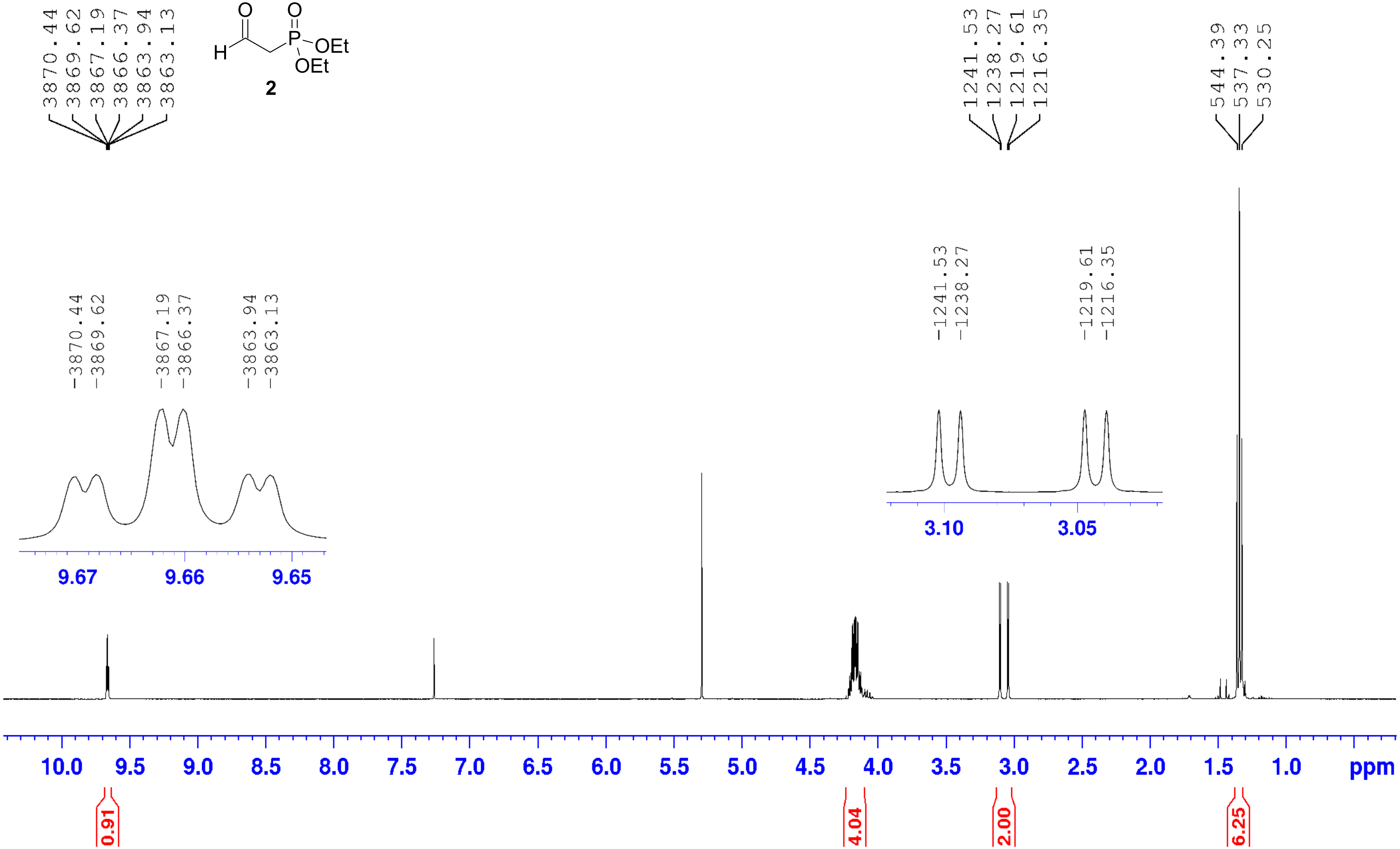

^{31}P NMR (162.03 MHz, $CDCl_3$) **of diethyl 2-oxoethylphosphonate (2):**

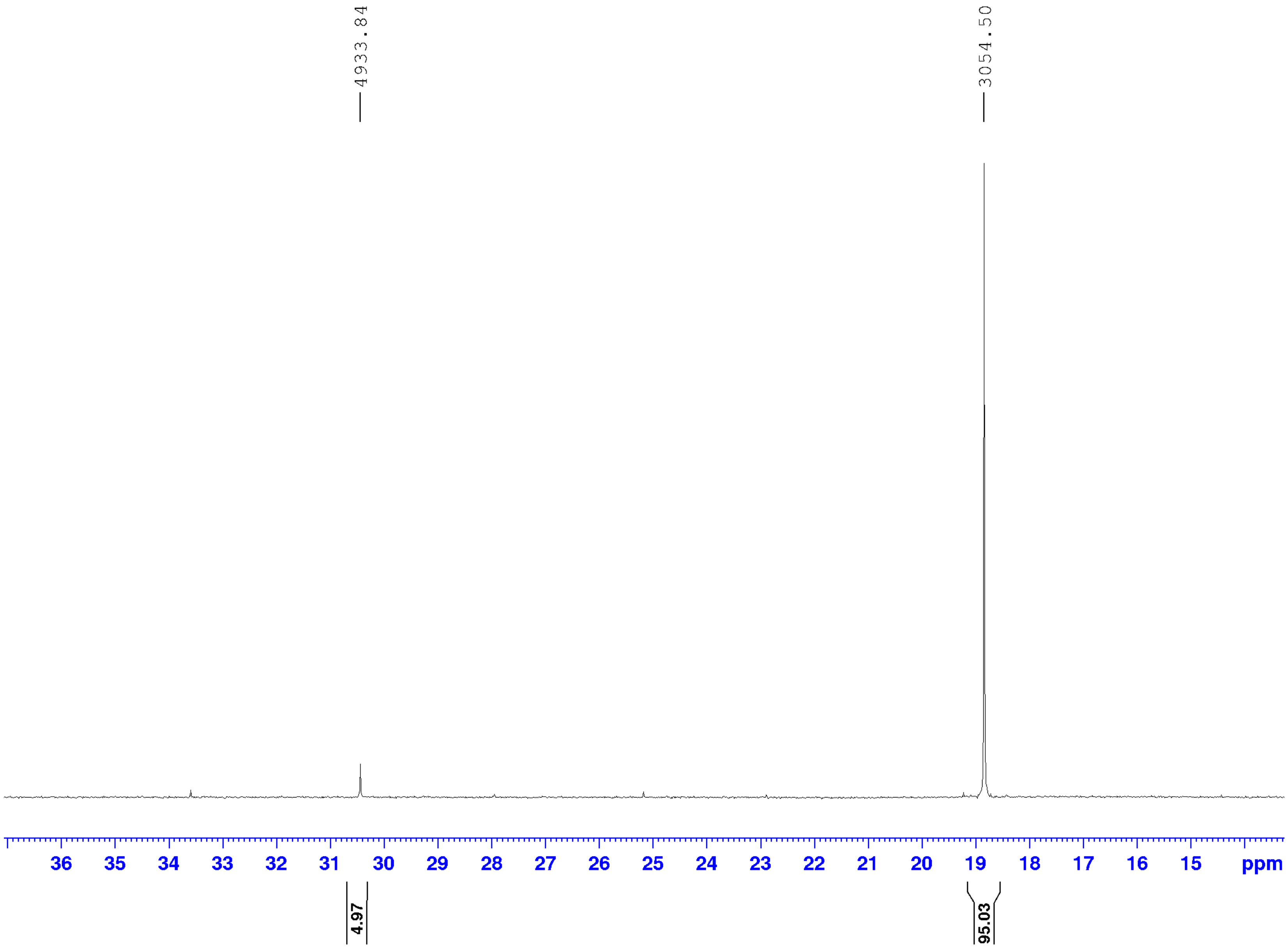

[1]**H NMR** (400.27 MHz, D_2O) **of phosphonoacetaldehyde and its hydrate as disodium salts (3 and 4):**

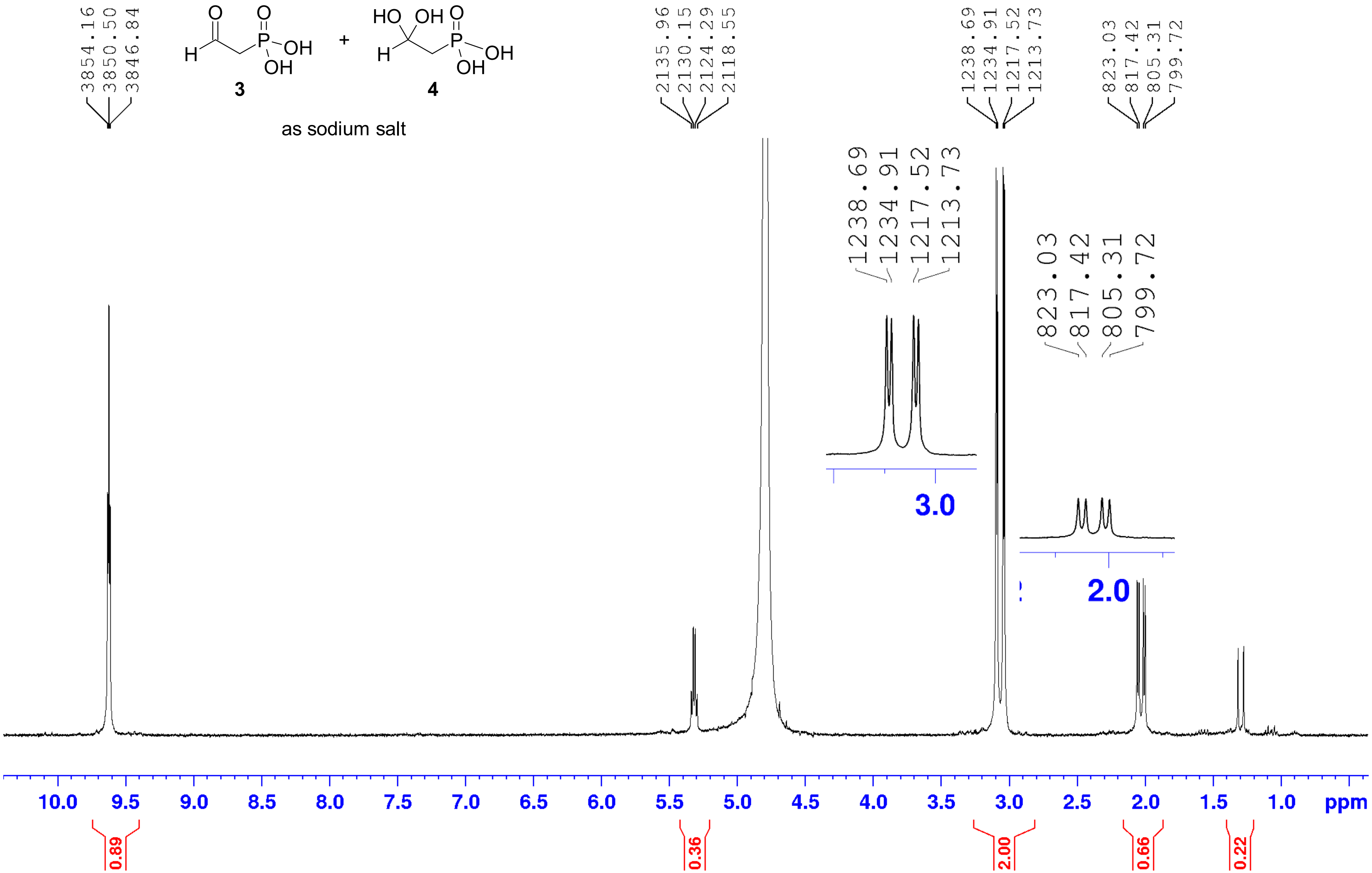

^{31}P NMR (162.03 MHz, D_2O) **of phosphonoacetaldehyde and its hydrate as disodium salts (3 and 4):**

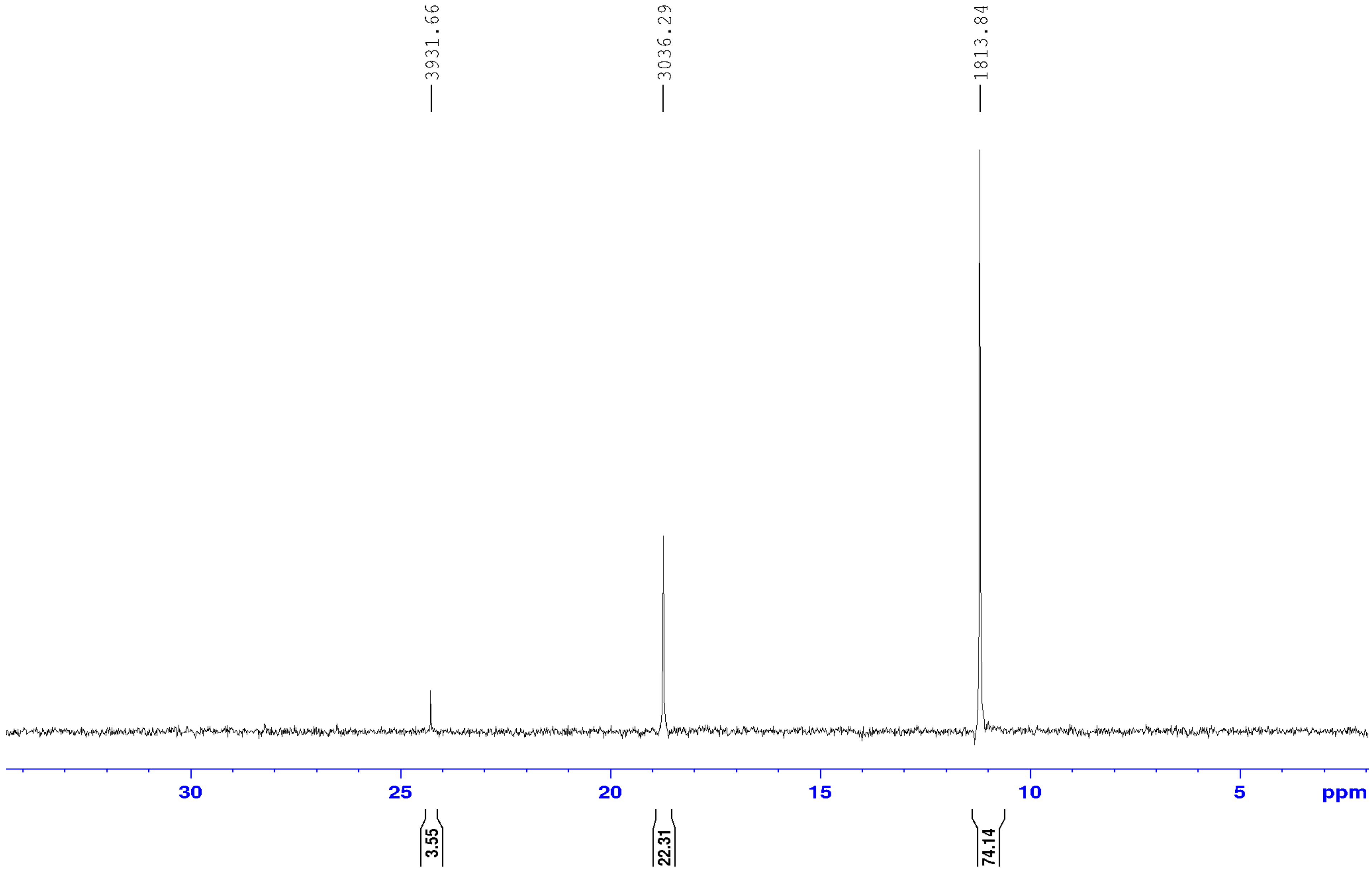

^{13}C NMR (176.13 MHz, D_2O) **of phosphonoacetaldehyde and its hydrate as disodium salts (3 and 4):**

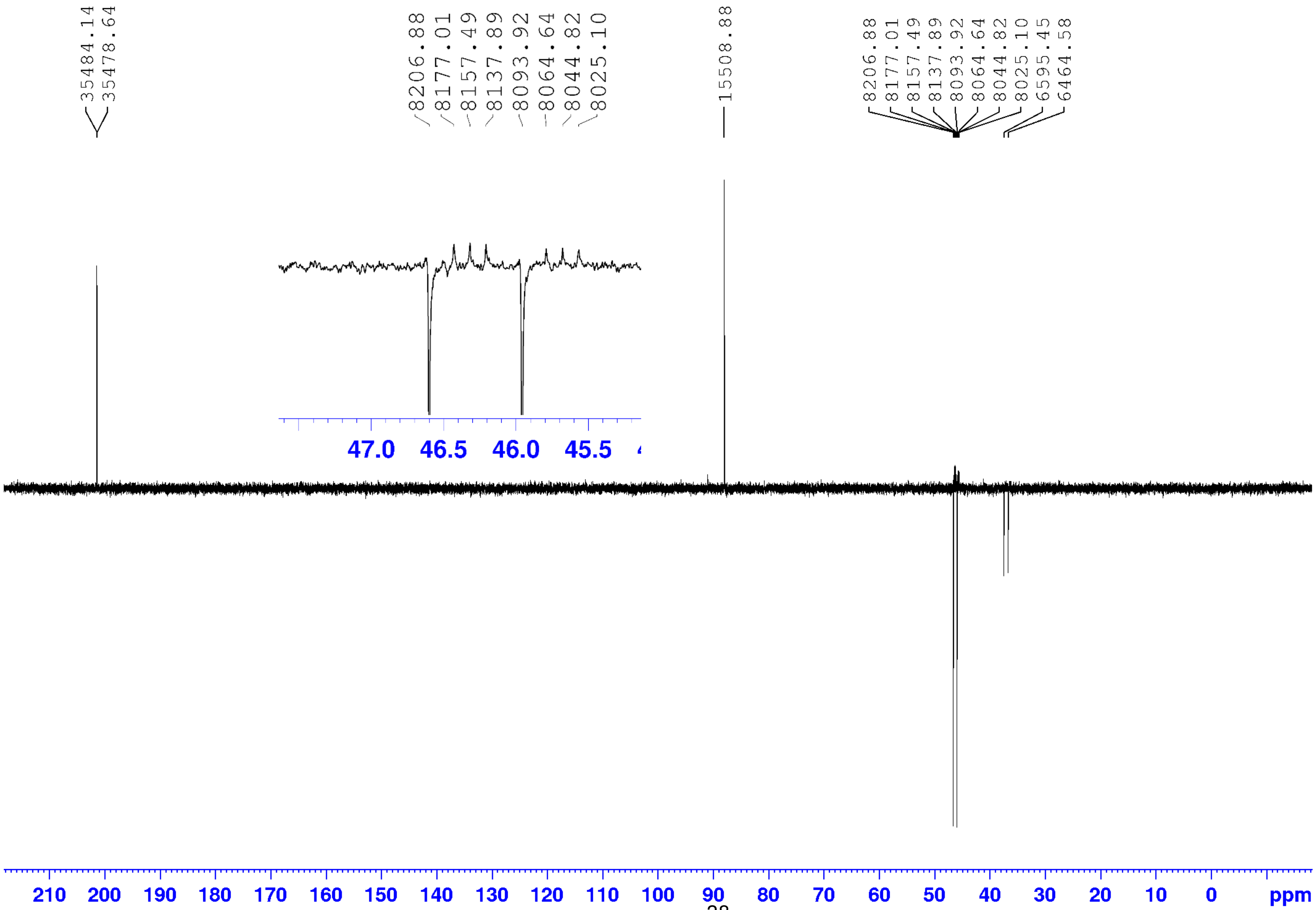

Supplementary References

Bock T, Müller R, Blankenfeldt W. Crystal structure of AibC, a reductase involved in alternative *de novo* isovaleryl coenzyme A biosynthesis in *Myxococcus xanthus*. Acta Crystallogr F Struct Biol Commun. 2016;72(8):652–8.

Born DA, Ulrich EC, Ju K-S, Peck SC, van der Donk WA, Drennan CL. Structural basis for methylphosphonate biosynthesis. Science. 2017;358(6368):1336–9.

DeLano WL. Pymol: an open-source molecular graphics tool. CCP4 Newsletter on Protein Crystallography. 2002;40:82–92.

Ishikawa K, Higashi N, Nakamura T, Matsuura T, Nakagawa A. The first crystal structure of L-threonine dehydrogenase. J Mol Biol. 2007;366(3):857–67.

van Kempen M, Kim SS, Tumescheit C, Mirdita M, Lee J, Gilchrist CLM, et al. Fast and accurate protein structure search with Foldseek. Nat Biotechnol. 2024;42(2):243–6.

Metcalf WW, Griffin BM, Cicchillo RM, Gao J, Janga SC, Cooke HA, et al. Synthesis of methylphosphonic acid by marine microbes: a source for methane in the aerobic ocean. Science (1979). 2012;337(6098):1104–7.

Naguib M, Feldman N, Zarodkiewicz P, Shropshire H, Biamis C, El-Halfawy OM, et al. An evolutionary conserved detoxification system for membrane lipid–derived peroxyl radicals in Gram-negative bacteria. PLoS Biol. 2022;20(5):e3001610.

Shao Z, Blodgett JAV, Circello BT, Eliot AC, Woodyer R, Li G, et al. Biosynthesis of 2-hydroxyethylphosphonate, an unexpected intermediate common to multiple phosphonate biosynthetic pathways. J Biol Chem. 2008;283(34):23161–8.

Thomas LM, Harper AR, Miner WA, Ajufo HO, Branscum KM, Kao L, et al. Structure of *Escherichia coli* AdhP (ethanol-inducible dehydrogenase) with bound NAD. Acta Crystallogr Sect F Struct Biol Cryst Commun. 2013;69(7):730–2.

Zangelmi E, Ruffolo F, Dinhof T, Gerdol M, Malatesta M, Chin JP, et al. Deciphering the role of recurrent FAD-dependent enzymes in bacterial phosphonate catabolism. IScience. 2023;26:108108.

Zangelmi E, Stanković T, Malatesta M, Acquotti D, Pallitsch K, Peracchi A. Discovery of a new, recurrent enzyme in bacterial phosphonate degradation: (*R*)-1-hydroxy-2-aminoethylphosphonate ammonia-lyase. Biochemistry. 2021;60(15):1214–25.

Zhang Y, Pham TM, Kayrouz C, Ju KS. Biosynthesis of argolaphos illuminates the unusual biochemical origins of aminomethylphosphonate and Nϵ-hydroxyarginine containing natural products. J Am Chem Soc. 2022;144(22):9634–44.

Zhou Y, Wei Y, Nanjaraj Urs AN, Lin L, Xu T, Hu Y, et al. Identification and characterization of a new sulfoacetaldehyde reductase from the human gut bacterium *Bifidobacterium kashiwanohense*. Biosci Rep. 2019;39(6):BSR20190715.
